# Manual and semi-automatic determination of elbow angle-independent parameters for a model of the *biceps brachii* distal tendon based on ultrasonic imaging

**DOI:** 10.1371/journal.pone.0275128

**Published:** 2022-10-06

**Authors:** Malte Mechtenberg, Nils Grimmelsmann, Hanno Gerd Meyer, Axel Schneider

**Affiliations:** Biomechatronics and Embedded Systems Group, Bielefeld University of Applied Sciences, Bielefeld, NRW, Germany; Toronto Rehabilitation Institute - UHN, CANADA

## Abstract

Tendons consist of passive soft tissue with non linear material properties. They play a key role in force transmission from muscle to skeletal structure. The properties of tendons have been extensively examined *in vitro*. In this work, a non linear model of the distal *biceps brachii* tendon was parameterized based on measurements of myotendinous junction displacements *in vivo* at different load forces and elbow angles. The myotendinous junction displacement was extracted from ultrasound B-mode images within an experimental setup which also allowed for the retrieval of the exerted load forces as well as the elbow joint angles. To quantify the myotendinous junction movement based on visual features from ultrasound images, a manual and an automatic method were developed. The performance of both methods was compared. By means of exemplary data from three subjects, reliable fits of the tendon model were achieved. Further, different aspects of the non linear tendon model generated in this way could be reconciled with individual experiments from literature.

## Introduction

The prediction of human elbow joint movements can be achieved by means of biomechanical models of the skeleton, soft tissue, and muscles. Muscle forces are actively generated by muscle contraction, and transmitted to bones via tendons. The force generated by a muscle depends on its contraction velocity, length, and neural activation. The neural activation is usually measured indirectly in the form of surface electromyograms (sEMGs). The contraction velocity and length of the muscle can then be estimated based on biomechanical models which make use of sEMG signals. In case of a musculoskeletal model including tendons, the muscle length depends on the skeletal geometry and tendon length. The length of a tendon depends, among other, non linearly on the exerted force (elasticity) [1]. In several *in vitro* experiments the elastic properties of mammalian tendon tissue have been measured [1–3], but data on the distal *biceps brachii* tendon is sparse [4, 5]. Further, for *in vivo* elbow movement prediction, an *in vivo* method to access biomechanical tendon parameters is preferable, as *in vitro* experiments with tendons of living subjects requires interventions in the body. *In vivo* approaches to analyze the elastic tendon properties are either invasive by implanting sensors into the tissue or non-invasive by monitoring the tendon shortening by medical imaging and tendon force estimation based on external force measurements [6]. In this context, ultrasonography can be used as an imaging technique [3, 7–10]. For the upper arm flexor muscles there are currently only a few studies on *in vivo* tendon properties as opposed to those studies which focus on tendons of muscles in lower extremities used for walking [11]. The former upper arm studies focus on the distal *biceps brachii* tendon [9, 10] but with an emphasis on the analysis of linear stiffness parameters for medical applications.

As stated above a model, which predicts the elbow movement, depends on the tendon length. Therefore, the behavior of the *biceps brachii* tendon is of interest, as the *biceps brachii* is a key contributing muscle in elbow flexion [12, 13]. Therefore, in this work, the goal was to obtain a configurable model for the distal *biceps brachii* tendon which reflects the non linear stiffness material properties of the tendon and can be used in conjunction with a muscle model for model-based elbow movement prediction.

In order to obtain stiffness model parameters for living subjects ideally the tendon length over tendon force would be measured. Here, the displacement of the distal *biceps brachii* myotendinous junction parallel to the humerus was observed by means of ultrasonic imaging at different loads, for different effective lever arms (due to varying joint angles) and while the subject maintained a fully supinated hand. As the tendon load could not be measured directly, it had to be estimated based on a skeletal model. An abstract skeletal model including a simplified *biceps brachii* tendon path was used to predict the tendon force corresponding to the force measured at the wrist for each elbow angle. The tendon displacement parallel to the *humerus* was extracted from the ultrasonography images. For this purpose, a manual and an algorithmic method were used. The algorithmic method was compared with the manual method for data extraction. It was found that the presented algorithmic method was partially not applicable resulting in an semi-automatic data extraction. However, the semi-automatic extraction greatly reduced the effort of feature extraction.

In the following, a general overview on mammalian tendon properties is given before a non linear tendon model is introduced. The presented stiffness model was fitted onto the manually and (semi-) automatically extracted tendon displacement dependent on the predicted tendon force.

## Mammalian tendon modeling

In general, tendons connect skeletal muscles to bones. Tendons insert into a bone via the *enthesis* and are connected to the muscle via the *aponeurosis*. The whole junction complex between muscle and *aponeurosis* is called myotendionous junction. In case of the distal *biceps brachii* myotendinous junction the *aponeurosis* additionally connects the *biceps brachii* with the forearm fascia [14].

**Fig 1:**
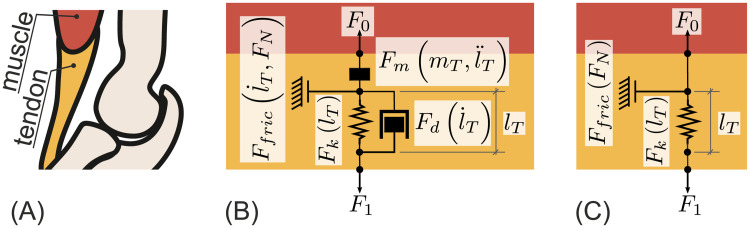
(A) Pictogram of the *biceps brachii* elbow flexor and its distal tendon with a mechanical model of the distal tendon in dynamic (B) and static conditions (C). (A) Pictogram of the *biceps brachii* (in red) and its lower tendon (in orange) attached to the *radius*. (B) A general tendon model including the tendon weight *m*_*T*_, non linear stiffness *F*_*k*_(Δ*l*), non linear damping $F_{d}\left({\dot{l}}_{T}\right)$, and friction to surrounding tissue $F_{firc}\left({\dot{l}}_{T},F_{N}\right)$. (C) This model is obtained by applying static conditions to the model shown in (B).

The role of a tendon in joint dynamics is determined by the geometry and material parameters [15]. Independent of the role, each tendon is understood to have elastic, movement damping properties [16]. Additionally creepage [17] and friction with the surroundings [18] were observed. A tendon model covering the aforementioned properties (except creepage) is shown in Fig 1B. The model in Fig 1B is composed of principal mechanical units.

If static conditions where assumed, motion-dependent, i.e. also time-dependent, properties would have no influence on the behavior of the presented model. If the assumption of static conditions is applied the tendon model depicted in Fig 1B is reduced to the elastic tendon properties and friction, as ${\dot{l}}_{T}={\ddot{l}}_{T}=0$ (Fig 1C). Furthermore in this work, friction is assumed to be negligible *F*_*fric*_ ≈ 0. As a consequence, only the elastic property of the tendon is further discussed. Elasticity is described as a force resisting changes in length. This is modeled by a function *F*(*Δl*). Where

$$\begin{array}{c} \Delta l=l-l_{0} \end{array}$$ (1)

is the difference between the actual length *l* and the slack length *l*_0_. Usually, elastic properties are expressed as force over strain where strain is defined as $\in =\frac{\Delta l}{l_{0}}$. This implies knowledge on the slack length *l*_0_. The slack length of a tendon in *in vivo* conditions is not necessarily known. Depending on the tendon in question and the experimental equipment that is available, it is likely that the slack length *l*_0_ has to be guessed. This work presents a method for myotendinous junction displacement estimation *in vivo* in which Δ*l* is measured. Therefore, the tendon has not to be modeled based on its resting (slack) length which is normally not available *in vivo*.

### Nonlinear static mammalian tendon properties

**Fig 2:**
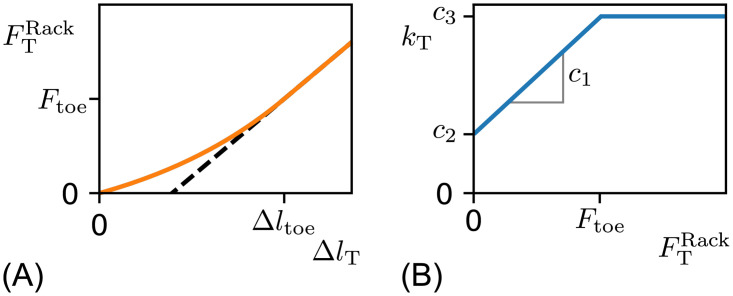
(A) Exponential elastic tendon lengthening model and its (B) stiffness as function of tendon force. In (A) the tendon force is shown over the change in tendon length in orange. As described in Eq (10). The linear force length relationship is extended with a dashed line. In (B) the stiffness *k*_T_ of the exponential tendon model is shown over the tendon force $F_{\text{T}}^{\text{Rack}}$. The tendon model parameters are marked in the plot. The non linear stiffness is configured by (*c*_1_ and *c*_2_). As shown in Eq (6) *c*_1_ is the change in stiffness *k*_T_ over a unit change in *F*. the parameter *c*_2_ is the stiffness at $F_{\text{T}}^{\text{Rack}}=0$. At the force *F*_toe_, the model continuously transitions into a linear stiffness function.

Mammalian tendons, which consist of collagen structures, barely resist shortening therefore,

$$\begin{array}{c} F_{\text{T}}(\Delta l_{\text{T}})=0\;\forall \;\Delta l_{\text{T}}\leq 0 \end{array}$$ (2)

is assumed. The tendon resists lengthening in a non linear behavior, as demonstrated in Fig 2A. This behavior is due to the collagen structure in which the collagen strains do engage at different lengths [15]. When the collagen structure is fully engaged the elastic behavior becomes linear. The tendon length at this state is called Δ*l*_toe_. The region 0 ≤ Δ*l*_T_ < = Δ*l*_toe_ is referred to as toe region as defined by [1]. If the tendon is at Δ*l*_T_ = 0, it is at its slack length, the length at which there is virtually no resistance to lengthening.

$$\begin{array}{c} F_{\text{T}}(\Delta l_{\text{T}})>0\;\forall \;\Delta l_{\text{T}}>0 \end{array}$$ (3)

For modeling the tendon force in relation to changes in length there are two major models. The first is a polynomial tendon model as described by Buchanan et al. [19]. The second is an exponential tendon stiffness model (see Eq (10)). The exponential tendon model describes a linear relationship between stiffness and force within the toe region as observed by Rack and Westbury [2]. The polynomial model as described by Buchanan does not have this property. Therefore, the exponential model is used throughout this work.

#### Tendon model with exponential force length relation by Rack and Westbury

A linear force stiffness relationship, as described by Rack and Westbury [2], can be reached by an exponential force strain model. In general the stiffness of a material is defined as

$$\begin{array}{c} \frac{\partial F}{\partial l}=k. \end{array}$$ (4)

Rack and Westbury could show, based on measurements of the cat *soleus* tendon, that the stiffness of a mammalian tendon *k*_T_ depends linearly on the applied force [2].

$$\begin{array}{c} k_{\text{T}}=\frac{{\partial F}_{\text{T}}^{\text{Rack}}}{\partial \Delta l_{\text{T}}}=c_{1}\cdot F_{\text{T}}^{\text{Rack}}+c_{2} \end{array}$$ (5)

Proske and Morgan concluded that the linear stiffness behaviour of Eq (5) is only valid in the toe region and assumed that the tendon stiffness beyond the toe region is constant [20], thus leading to a linear force strain behaviour beyond the toe region (Fig 2A). Based on these findings, the following differential equation models the stiffness for $F_{T}^{\text{Rack}}\geq 0$ during the lengthening of the tendon (Δ*l*_T_ ≥ 0).

$$\begin{array}{c} k_{\text{T}}=\frac{{\partial F}_{\text{T}}^{\text{Rack}}}{\partial \Delta l_{\text{T}}}=\{\begin{array}{cc} c_{1}\cdot F_{\text{T}}^{\text{Rack}}+c_{2} & ,0\leq F_{\text{T}}^{\text{Rack}}\leq F_{\text{toe}}\\ c_{1}\cdot F_{\text{toe}}+c_{2} & ,F_{\text{toe}}<F_{\text{T}}^{\text{Rack}} \end{array} \end{array}$$ (6)

Solving the differential equation for each case leads to the following expression, if *c*_2_ ≠ 0 is assumed.

$$\begin{array}{c} \begin{array}{cc} & F_{\text{T}}^{\text{Rack}}(\Delta l_{\text{T}})\\ & =\{\begin{array}{cc} -\frac{c_{2}}{c_{1}}+A_{1}\cdot e^{c_{1}\cdot \Delta l_{\text{T}}} & ,0\leq \Delta l_{\text{T}}\leq \Delta l_{\text{toe}}\\ \underset{m}{\underset{︸}{\left[c_{1}\cdot F_{\text{T}}^{\text{Rack}}(\Delta l_{\text{toe}})+c_{2}\right]}}\Delta l_{\text{T}}+A_{2} & ,\Delta l_{\text{toe}}<\Delta l_{\text{T}} \end{array} \end{array} \end{array}$$ (7)

Where Δ*l*_toe_ is the change in tendon length at which the toe region ends (see Fig 2A). *A*_1_ and *A*_2_ are coordinates in the solution space of Eq (7), which also contains physically implausible solutions. In order to gain a physically feasible solution, the condition from Eq (2) is applied and continuity of $F_{\text{T}}^{\text{Rack}}$ for Δ*l*_T_ > 0 is assumed. This results in the following, unique solution.

$$\begin{array}{c} A_{1}=\frac{c_{2}}{c_{1}} \end{array}$$ (8)

$$\begin{array}{c} A_{2}=[1-c_{1}\Delta l_{\text{toe}}]F_{\text{T}}^{\text{Rack}}(\Delta l_{\text{toe}})-c_{2}\Delta l_{\text{toe}} \end{array}$$ (9)

The substitution of *A*_1_ and *A*_2_ in Eq (7) leads to the following model description.

$$\begin{array}{c} \begin{array}{c} F_{\text{T}}^{\text{Rack}}\left(\Delta l_{\text{T}};\vec{\rho }\;\right)=\{\begin{array}{cc} \frac{c_{2}}{c_{1}}[e^{c_{1}\cdot \Delta l_{\text{T}}}-1] & ,0\leq \Delta l_{\text{T}}\leq \Delta l_{\text{toe}}\\ m\left(\vec{\rho }\;\right)\cdot \Delta l_{\text{T}}+A_{2}\left(\vec{\rho }\;\right) & ,\Delta l_{\text{toe}}<\Delta l_{\text{T}} \end{array} \end{array} \end{array}$$ (10)

Here, the tendon parameters are included explicitly in the function definition as parameter vector $\vec{\rho }$.

$$\begin{array}{ccc} F_{\text{T}}^{\text{Rack}}\left(\Delta l_{\text{T}}\;;\vec{\rho }\;\right) & \; & ,\text{where}\;\vec{\rho }=[\begin{array}{c} c_{1}\\ c_{2}\\ \Delta l_{\text{toe}} \end{array}] \end{array}$$ (11)

Eq (10) is not defined for compression (Δ*l*_T_ < 0). It is widely assumed that the tendon resists with nearly no force to compression [19, 21]. This compression behaviour, however, is not needed in this study as the tendon will only be observed in extension. Due to the type of data acquired in this study, the inverse of Eq (10) is needed.

$$\begin{array}{c} \begin{array}{cc} & \Delta l_{\text{T}}^{\text{Rack}}(F_{\text{T}};\vec{\rho })\\ & =\{\begin{array}{cc} \frac{\text{ln}(1+\frac{c_{1}}{c_{2}}\cdot F_{\text{T}})}{c_{1}} & ,0\leq F_{\text{T}}\leq \frac{c_{2}}{c_{1}}[e^{\Delta l_{\text{toe}}\cdot c_{1}}-1]\\ \frac{F_{\text{T}}-A_{2}(\vec{\rho })}{m(\vec{\rho })} & ,\frac{c_{2}}{c_{1}}[e^{\Delta l_{\text{toe}}\cdot c_{1}}-1]<F_{\text{T}} \end{array} \end{array} \end{array}$$ (12)

Eq (12) is used for the tendon model that was used to represent the experimental data as described in the following section.

## Methods for tendon modelling based on in vivo measurements

Experiments were conducted with 5 subjects from which 3 are shown in this study. All subjects where recruited by call for participation and gave written informed consent to the pseudo anonymous usage of the recorded data. All subjects that responded to the call for participation and stated that they had no related injuries where accepted for the study. The study was conducted according to the ethical guidelines of the German Society for Psychology (DGPs) and the German Psychologists Association (BdP), and approved by the Ethics Committee of the University of Bielefeld (EUB 2017–156 02.08.2017). The experiment data is published in a zenodo data repository [22].

The displacement of the lower *biceps brachii* myotendinous junction Δ*L*_*mt*_ was measured over 7 elbow angles Θ and over 6 levels of desired force *F*_w_ for each subject. The force was measured at the subjects wrist in isometric conditions.

$$\begin{array}{c} \begin{array}{cc} \Delta L_{mt}\left(\Theta ,F_{\text{w}}\right)\; & \text{where}\\ \Theta & \in \{90{\;}^{\circ },75{\;}^{\circ },60{\;}^{\circ },45{\;}^{\circ },30{\;}^{\circ },15{\;}^{\circ },0{\;}^{\circ }\}\\ F_{\text{w}} & \in \{5\;\text{N},10\;\text{N},15\;\text{N},30\;\text{N},75\;\text{N},120\;\text{N}\} \end{array} \end{array}$$ (13)

**Fig 3:**
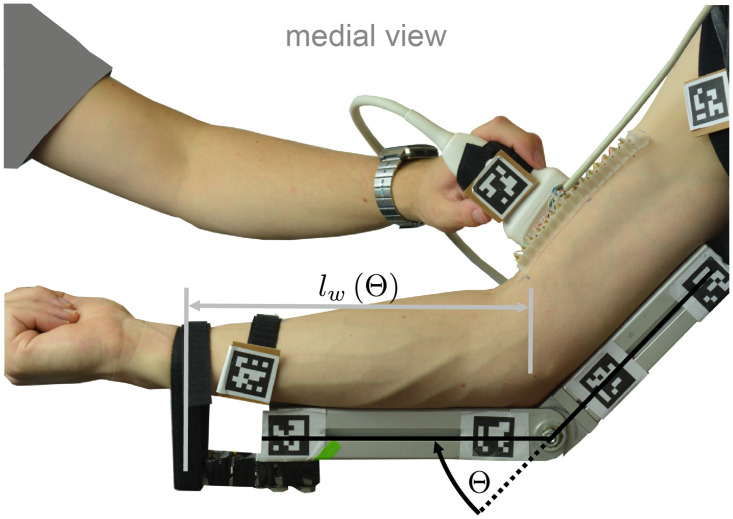
A medial view on the experimental setup. The distance *l*_*w*_(Θ) between the elbow and the wrist strap, which mechanically connects wrist and load cell, is marked with gray lines. The distance *l*_*w*_ depends on the elbow angle Θ, which is shown in black. A change in joint angle is constrained by the depicted construction of aluminium profiles. If the screw in the hinge joint of the profile structure is loosened, the joint angle can be adjusted.

During the experiment, the right arm of a subject was positioned on a support rack, as shown in Fig 3. The support rack was used to keep the elbow angle constant during the experiment. A force sensor consisting of a load cell and strain gauges in half bridge configuration was used to measure the force at the wrist $F_{\text{w}}^{\text{raw}}$, while the hand was fully supinated. A hook-and-loop strap mechanically connected the wrist to the load cell. The lengthening of the wrist strap was assumed to be negligible in terms of changes in Θ (linear stiffness of the wrist strap is $39.39\;\frac{\text{N}}{\text{mm}}$ at a preload force of 15N). The strain gauges were processed with a bridge measurement module (NI-9237, National Instruments Corp., Austin, TX, USA) and $F_{\text{w}}^{\text{raw}}$ was logged via LabVIEW (National Instruments Corp.) at a sampling frequency of *f*_*F*_ = 1kHz. The measured force was offset by the wrist strap preload at the beginning of the experiment, filtered with a moving average filter over 40 samples and fed back to the subject via a virtual gauge displayed on a computer screen using LabVIEW.

The myotendinous junction was observed using an ultrasonic device in B-mode. The ultrasonic transducer was handled by an experienced operator. As indicated in Fig 3, the tendon displacement together with the wrist force was measured in three steps.

1. In the unloaded muscle state (no wrist force) a video capture of the ultrasonic B-mode image was started. (*t*_0_ = 0s)
2. The subject was instructed to flex the right arm such that the desired force level could be measured at the wrist (feedback via computer screen). (*t*_1_ = 2s)
3. The B-mode video capture was stopped and then the subject was instructed to relax again. (*t*_2_ = 8.5s)
4. At least 20 seconds of rest.

These steps were initiated by beeping sounds at the given times *t* and repeated for each desired force level *F*_w_ at each elbow angle Θ. For each subject the experiment started at an elbow angle of Θ = 90^∘^. First, the maximal voluntary contraction force was measured without ultrasonography imaging. Then each desired force level was measured three times in ascending order before the elbow angle was reduced by 15^∘^ (note Eq (13)).

Later, the movement of the myotendinous junction was extracted from the B-mode video captures.

Additionally, distances between anatomic landmarks of the arm were measured with a tailor tape measure (S1 Table). These distances were used as the basis of a skeletal arm model (cmp. Eq (37)). With this model, the tendon properties were determined by means of a fitting approach. Here, it was assumed, that any observed movement of the myotendinous junction parallel to the *humerus* is attributed to the lengthening of the lower tendon of the *biceps brachii* muscle during contraction. Using force and shortening information, a local tendon stiffness for a given elbow angle Θ can be estimated or a global characteristic curve which is valid for several elbow angles can be constructed. The latter was the goal of this work.

### In vivo measurement of the tendon displacement

**Fig 4:**
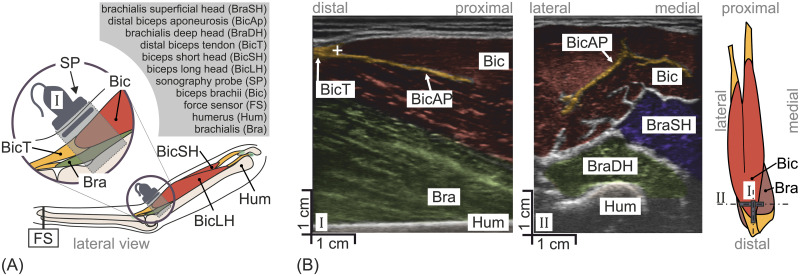
**In (A) the lateral view of the experimental setup in form of an anatomical sketch is shown**. An ultrasonic probe (SP) is used to image a cross-section of the distal part of the upper arm. The cross-section contains the distal tendon of the *biceps brachii* (BicT). Simultaneously, the force generated by the arm is measured with a force sensor (FS) near the wrist (cmp. Fig 3). The *biceps brachii* (Bic), its long head (BicLH), its short head (BicSH), *brachialis* (Bra) and *humerus* (Hum) are also labeled. **In (B) the ultrasonic images of two orthogonal cross-sections are depicted.** The orientation of both imaging planes are marked in a sketch on the right side, which shows the anterior perspective on the Bic and Bra. Orientation I was the only imaging plane used during the presented study (cmp. (A)). The structures of the Bic are marked in red. The junction of Bic and BicT was observable in this view (white cross) as well as part of the *aponeurosis* (BicAP). Imaging plane II is orthogonal to imaging plane I. The furcation (wing-like structure) of the *aponeurosis* was observable (marked in orange) as well as different parts of the Bra muscle group—the *brachialis deep head* (BraDH) in green and the *brachialis* superficial head (BraSH) in blue.

**Fig 5:**
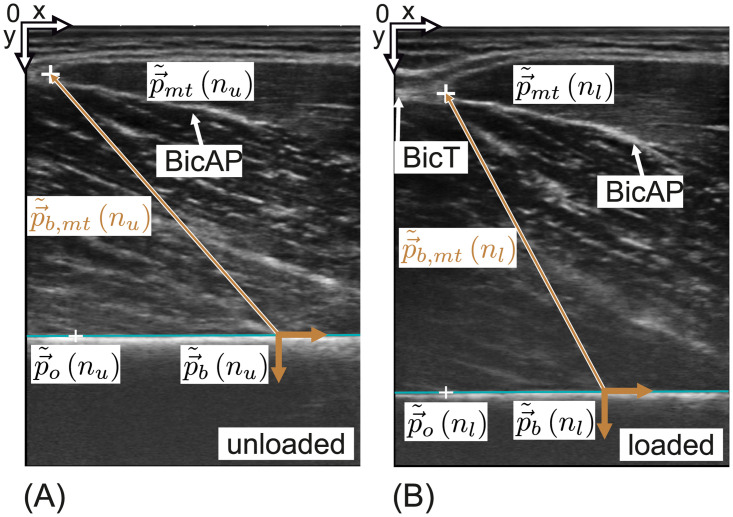
Ultrasonic images of the myotendinous junction (white cross) in the parasagittal plane for the (A) unloaded and (B) loaded case (cmp. Fig 4A, orientation I) are shown. (A) shows the unloaded state *n*_*u*_ (*F*_*w*_ = 0) and (B) the loaded state *n*_*l*_. Reference points ${\tilde{\vec{p}}}_{b}$ on the *humerus* and ${\tilde{\vec{p}}}_{mt}$ on the myotendinous junction are marked in the frame of each state. The reference points were used to calculate the position of the myotendinous junction ${\tilde{\vec{p}}}_{b,mt}$ relative to the bone local coordinate system (brown). To calculate the bone surface orientation within the frame, a second marker ${\tilde{\vec{p}}}_{o}$ was placed arbitrarily on the bone surface. That is ${\tilde{\vec{p}}}_{o}\left(n_{u}\right)$ for the unloaded state and ${\tilde{\vec{p}}}_{o}\left(n_{l}\right)$ for the loaded state. The bone line was set such that it included both bone points ${\tilde{\vec{p}}}_{o}$ and ${\tilde{\vec{p}}}_{b}$. The bone line is drawn in turquoise for each frame.

In order to quantify the displacement of the lower *biceps brachii* myotendinous junction, ultrasonic video images of the anatomical structure were recorded with an E1 ultrasound device equipped with the 50 mm linear ultrasonic array transducer L741 (SonoScape Medical Corp. Shen Zhen, P.R. China) in B-mode at a frequency spectrum of 9.5MHz to 12.2MHz. The linear array was chosen as its field of view allows safe observations of the myotendinous junction during the complete shortening process up to the maximal voluntary contraction. The intensity in a B-mode image corresponds to the amplitude of reflected acoustic waves. Reflections occur at tissue changes due to varying material properties. A homogeneous tissue section would appear in black, in contrary to inhomogeneous tissue sections or junctions between two homogeneous sections. Fig 4A shows the lateral view of the measurement setup as an anatomical sketch with an orientation of the linear array (SP) that corresponds to the imaging orientation in Fig 4B (left, marked I). In Fig 4B the actual two ultrasonic images of the myotendinous junction in two imaging orientations I (Fig 4B, left) and II (Fig 4B, middle) are given. To further illustrate the position and orientation (imaging plane I or II) of the linear ultrasonic array, an anatomical sketch of the *biceps brachii* and *brachialis* from an anterior perspective is given on the right side of Fig 4B. Imaging plane I (left side of Fig 4B) represents the parasagittal plane crossing the *humerus* (Hum) and the *aponeurosis* (BicAP). The *aponeurosis* connects the tendon (BicT) to the muscle (cmp. Fig 4A). Here, each muscle fiber’s terminal *Z disk* is connected to the tendon collagen structure [23]. Due to the change in material properties, the *aponeurosis* appears as a distinct feature in the ultrasonic image. As the muscle fibers gradually terminate, the muscle belly flattens out. A distinct region is identified where the *aponeurosis* emerges from the main tendon structure (furcation of BicT ranging into the *biceps* Bic). This point is referred to as ${\vec{p}}_{\text{mt}}$, and is marked by a white cross in Figs 4B and 5. Contraction of the muscle leads to movement of that point. Due to the anisotropic structure of skeletal muscle, the *biceps brachii* can be discriminated from the *brachialis* muscle group (Bra). The cross-sectional view in imaging plane II allows a clear distinction of the individual muscles, as the the muscle structure in the abaxial plane is mostly homogeneous as opposed to the tissue transitions.

During the experiment the ultrasonography operator focused on maintaining the imaging plane I (cmp. Fig 4B). Hence, the operator had to constantly compensate changes of the probe orientation, induced by skin surface changes during contraction. The operator focused on maintaining the same reference points marked by *brachialis* insertion into the *humerus*, as well as the myotendionous junction within the frame. If this was not possible, the operator instructed a repetition of the measurement.

#### Manual extraction of the myotendinous junction displacement

**Fig 6:**
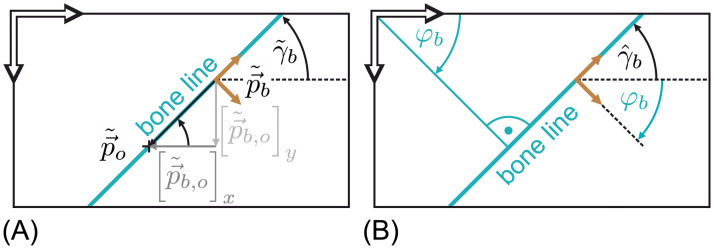
Bone line angle calculation in case of (A) the manual and (B) the automatic data extraction. A sketch which demonstrates how the bone line angle was calculated during manual data extraction (${\tilde{\gamma }}_{b}$) and automatic data extraction (${\hat{\gamma }}_{b}$). (A) demonstrates the case of manual bone line labeling in which ${\tilde{\gamma }}_{b}$ was derived from the vector ${\tilde{\vec{p}}}_{b,o}$ as described in Eq (16). Analog (B) demonstrates how ${\hat{\gamma }}_{b}$ was derived from a bone line expressed in *Hough*-line parameters (cmp. Fig 9) as formulated in Eq (24).

In Fig 5 two B-mode frames in the parasagittal plane are shown (for reference compare with orientation of imaging plane I in Fig 4A). These frames were selected by an experienced ultrasonography operator who decided, based on the movement of the anatomical structures, when the steady states of the myotendinous junction movements were reached. The first steady state was the unloaded state whereas the second state was reached when the subject applied the instructed force level. Due to deformation of the tissue during contraction, the two frames show slightly different viewpoints. During the experiment, the ultrasonography operator chose a fixed reference point and made sure that it stayed within the frame. As reference points, insertion points of *brachialis* fibres at the *humerus* were chosen as these are fixed within the coordinate system of the *humerus*. These points served as reference coordinate system. Fig 5 depicts the bone coordinate frame at that reference point in brown for the unloaded Fig 5A and loaded state Fig 5B. It is oriented such that the abscissa is congruent with the bone surface. The bone surface in the B-mode image is abstracted as a line (Fig 5, drawn in turquoise). The bone line was parametrized by means of of the orientation angle ${\tilde{\gamma }}_{b}$ (see Fig 6) and the bone reference point ${\tilde{\vec{p}}}_{b}$ (where the tilde symbol indicates that the respective quantity is linked to the manual data extraction). The orientation angle ${\tilde{\gamma }}_{b}$ was derived from the bone reference point ${\tilde{\vec{p}}}_{b}$ and a second marker ${\tilde{\vec{p}}}_{o}$. These parameters were manually marked for the unloaded state *n*_*u*_ and loaded state *n*_*l*_.

The point of the myotendinous junction ${\tilde{\vec{p}}}_{\text{mt}}$ was chosen where the *aponeurosis* crosses with an imaginary extension of the fascia in both states. The junction point was transformed into the reference coordinate system for each frame.

$$\begin{array}{c} \begin{array}{c} {\tilde{\vec{p}}}_{\text{b,mt}}\left(n_{k}\right)=R^{-1}({\tilde{\gamma }}_{b}\left(n_{k}\right))\cdot ({\tilde{\vec{p}}}_{\text{mt}}\left(n_{k}\right)-{\tilde{\vec{p}}}_{\text{b}}\left(n_{k}\right))\\ \text{with}\;k\in \{\text{u},\text{l}\} \end{array} \end{array}$$ (14)

Where ${\tilde{\gamma }}_{b}\left(n_{k}\right)$ is the bone line orientation angle relative to the abscissa of the bone local coordinate system. And **R**(•) is the rotation matrix for the bone local coordinate system. The bone local coordinate system is a left handed coordinate system. $R\left(•\right)\cdot \vec{\nu }$ rotates $\vec{\nu }$ in the clock wise direction for positive angles. Consequently **R**^−1^(•) and ${\tilde{\gamma }}_{b}\left(n_{k}\right)$ are described as follows.

$$\begin{array}{c} R^{-1}\left(•\right)=[\begin{array}{cc} \text{cos}(•) & \text{sin}(•)\\ -\text{sin}(•) & \text{cos}(•) \end{array}] \end{array}$$ (15)

$$\begin{array}{c} {\tilde{\gamma }}_{b}\left(n_{k}\right)=\text{arctan}(\frac{{\left[{\tilde{\vec{p}}}_{b,o}(n_{k})\right]}_{y}}{{\left[{\tilde{\vec{p}}}_{b,o}(n_{k})\right]}_{x}}) \end{array}$$ (16)

Where ${\tilde{\vec{p}}}_{b,o}={\tilde{\vec{p}}}_{o}-{\tilde{\vec{p}}}_{b}$ is the vector pointing from the bone reference point to the bone orientation marker as shown in Fig 6A. The operator [•]_*x*_ returns the *x* coordinate and [•]_*y*_ the *y* coordinate of a vector.

#### Calculation of myotendinous junction movement

After the myotendinous junction ${\tilde{\vec{p}}}_{\text{b,mt}}\left(n_{k}\right)$ was described in the bone local coordinate system, the tendon lengthening could be calculated for each frame. If the absolute position of the bone reference point in a skeleton model and the path of the tendon were known, the lengthening of the tendon could be calculated accurately. In this work, the lengthening of the tendon was assumed as the movement of the myotendinous junction from unloaded to the loaded state parallel to the bone line.

$$\begin{array}{c} \Delta {\tilde{\vec{p}}}_{\text{b,mt}}(\Theta ,F_{\text{w}})={\tilde{\vec{p}}}_{\text{b,mt}}\left(n_{\text{l}};\Theta ,F_{\text{w}}\right)-{\tilde{\vec{p}}}_{\text{b,mt}}\left(n_{\text{u}};\Theta ,F_{\text{w}}\right) \end{array}$$ (17)

$$\begin{array}{c} \Delta {\tilde{L}}_{\text{mt}}(\Theta ,F_{\text{w}})={\left[\Delta {\tilde{\vec{p}}}_{\text{b,mt}}(\Theta ,F_{\text{w}})\right]}_{x} \end{array}$$ (18)

**Fig 7:**
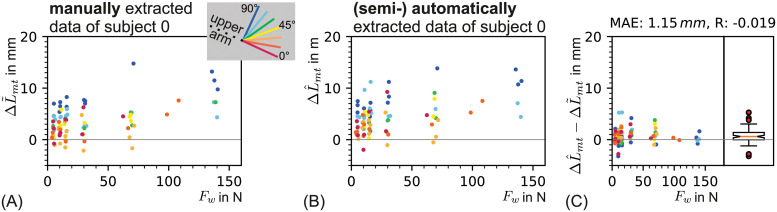
Tendon displacement over wrist force for all elbow angles is shwon in case of (A) manual and (B) (semi-) automatic data extraction, with (C) a comparison of both methods. The length of the junction movement parallel to the bone of subject 0 is shown for the manually extracted data $\Delta {\tilde{L}}_{mt}$ as depicted in (A) and for the automatically extracted data $\Delta {\hat{L}}_{mt}$ in (B). In both cases the mytendinous junction movement parallel to the bone is shown over the wrist force *F*_*w*_ for all elbow angles Θ (cmp. Eq (13)). The bone line angle as well as the evaluated frames are adopted from the manual evaluation as described in Eq (31). In (C) the difference between the automatically extracted data $\Delta {\hat{L}}_{mt}$ and the manually extracted data $\Delta {\tilde{L}}_{mt}$ of subject 0 is shown.

Data for $\Delta {\tilde{L}}_{\text{mt}}$ of subject 0 is presented as an example in Fig 7A. Later the quality of the extracted data is discussed and compared with a (semi-) automatic approach.

#### Computer aided extraction of the myotendinous junction displacement

In addition to the manual procedure for extracting the myotendinous displacement (see Eqs (17) and (18)) a computer-aided procedure was employed. The computer-assisted method allows a) to minimize any inaccuracies due to the experimenter’s subjective judgment during manual extraction of reference points, b) to ensure reproducibility of results, and c) to achieve higher throughput when processing large data sets.

**Fig 8:**
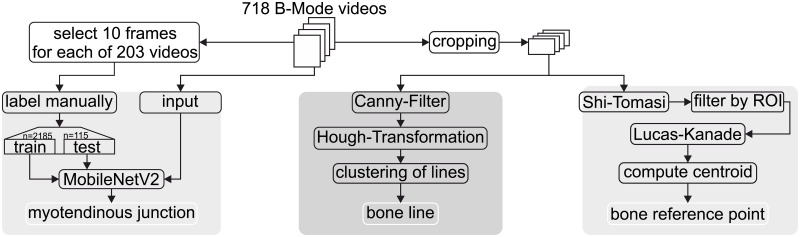
Block diagram for each computational method of feature extraction is shown. The computed features are from left to right: the mytendinous junction, bone line and bone reference point. The mytendinous junction is tracked with a neural network based on the *MobileNetV2* algorithm. The bone line is tracked via *Hough*-Line-Transformation. The bone reference point is tracked by a method based on the *Lucas-Kanade* algorithm.

In order to calculate the junction movement $\Delta {\hat{L}}_{\text{mt}}$, the computer-aided method extracts the myotendinous junction ${\hat{\vec{p}}}_{\text{mt}}$, the relative orientation of the bone $\hat{\gamma }$ and the bone reference point ${\hat{\vec{p}}}_{\text{b}}$ from B-mode ultrasound frames based on different algorithmic methods as depicted in Fig 8 and described in the following paragraphs. The hat symbol is used as annotation of measurement quantities related to the computer-aided feature extraction.

#### Detection of myotendinous junction using deep neural networks

In order to detect the myotendinous junction ${\hat{\vec{p}}}_{\text{mt}}$ from the data set of B-mode ultrasound frames, a deep learning approach using transfer learning was used (see Fig 8). Automated tracking of the myotendinous junction using convolutional neural networks has recently been shown to provide accurate results comparable to manual tracking methods [24, 25] and to be less error-prone than semi- and fully-automated myotendinous junction tracking approaches based on optic flow [26, 27] or block-matching algorithms [28, 29].

To generate and train a convolutional neural network capable of detecting the myotendinous junction ${\hat{\vec{p}}}_{\text{mt}}$ of the *biceps brachii* from ultrasound images, the markerless pose-estimation framework *DeepLabCut* [30] was used. The architecture of the underlying convolutional network is based on *MobileNetV2* [31] and was pretrained on the *ImageNet* data set by [32]. A training data set was generated via random selection of 203 video sequences from the data set of B-mode ultrasound images. From each individual video sequence 10 images were extracted in a randomly and temporally uniformly distributed manner resulting in a total of (*n* =) 2030 frames. In each of the extracted image frames, the myotendinous junction of the *biceps brachii* was labeled by hand, hence, serving as a ground truth. The labeled images were subsequently split into two data sets for use in training (*n*_*t*_ = 2185 frames) and evaluation (*n*_*e*_ = 115 frames). Training of the neural network was performed using the training data set with a batch size of 20 for 200000 iterations and *DeepLabCut*’s standard parameters. The performance of the neural network was subsequently evaluated via computation of the the mean average error (MAE) of the Euclidean distance between the predicted and the ground truth location of the myotendinous junction in the evaluation data set. This resulted in an MAE of 4.32 pixels corresponding to 0.50*mm*. After training, the neural network was used to automatically infer the location of the myotendinous junction ${\hat{\vec{p}}}_{\text{mt}}(n)$ from the individual video sequences of B-mode ultrasound images (hat symbol indicates computer-aided feature extraction).

#### Computation of the bone orientation

**Fig 9:**
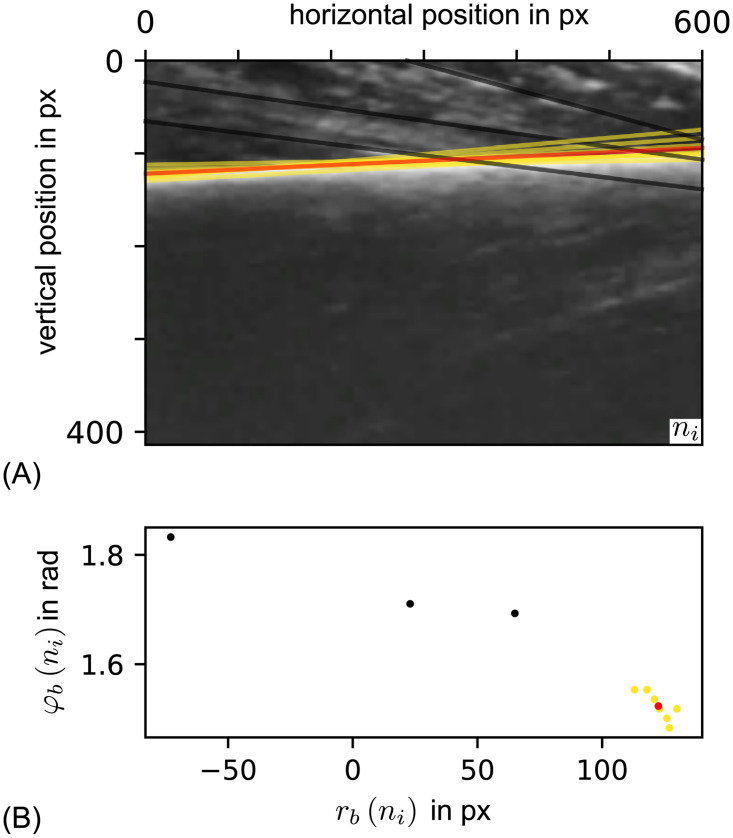
(A) Bone lines detected in a B-mode frame and (B) shown in parameter space. (A) is a frame of the cropped section of a B-mode video. The detected lines have the same color coding as in the scatter plot of the bone line parameter space. (B) The corresponding scatter plot of detected *Hough*-Lines in their parameter space. The biggest detected cluster is shown in yellow and its mean is shown in red. All other data points are black.

The bone orientation ${\tilde{\gamma }}_{b}$ relative to the *x*-axis was previously calculated from two manually selected points on the bone surface $\left({\tilde{\vec{p}}}_{b},{\tilde{\vec{p}}}_{o}\right)$. Due to the lack of explicitly definable features on the bone surface for these points in the B-mode frames, feature tracking analog to the myotendinous junction was not an option. The bone surface appears as a continuous feature in the B-mode image as depicted in Fig 5. However, the bone surface has a high contrast as compared to the surrounding muscle tissue. The transition from muscle tissue to bone can be abstracted as a line. Therefore, the B-mode image was filtered for edges and a line detection algorithm was applied. As the fibers in the muscle tissue also have a line characteristic, the B-mode image was cropped such that the risk of a line feature in the muscle tissue being detected as bone line was minimized. A block diagram of this process is shown in the middle section of Fig 8. In order to confine the algorithm to an image region relevant for the detection of the visual line separating muscle from bone tissue, each ultrasound image frame was cropped using the *FFMPEG* library [33] resulting in a size of (*w*_*c*_ =) 600 by (*h*_*c*_ =) 414 pixels of the cropped frame. (*x*_*c*_ = 320, *y*_*c*_ = 350) is the horizontal and vertical pixel position of the top left corner of the cropped region within the original frame (see Fig 9A). After cropping, the *Canny edge detection* algorithm ([34], as implemented in *OpenCV* [35]), was applied to each frame. This algorithm contains a four-stage process in which 2 of the 4 stages need to be parameterized. First, a noise reduction with a 5*x*5 *Gaussian* filter is applied. Second, the intensity gradient is computed. This process is parameterized by the aperture size (*a*_*s*_ = 3) and the norm used in gradient computation. Here, the computationally efficient *L*_1_ norm was used. Third, after gradient computation, a non-maximum suppression is applied. The result is an image in which the value of each pixel describes the gradient of an edge. Finally, in the fourth step, these edges are filtered by hysteresis thresholding. In this work, pixel with a gradient value above grad_max_ = 220 were accepted as edges. Pixel with a gradient value above grad_min_ = 50 were considered edges if they were connected to edges above grad_max_. All other pixel/edges were filtered out. The used parameters are shown in S2 Table.

The remaining edges were used during the *Hough*-transformation for line detection. The *Hough*-lines are defined by a vector $\vec{r}$ in cylindrical coordinates (*r*, *φ*). The vector has to be understood as a point on the described line $\vec{r}={\vec{l}}_{\text{b}}(h=0)$. Where ${\vec{l}}_{\text{b}}(h)$ is the parameter form of the bone line, which is defined as follows.

$$\begin{array}{c} {\vec{l}}_{\text{b}}(h)={\vec{m}}_{b}\cdot h+\vec{b} \end{array}$$ (19)

Furthermore, it is assumed that $\vec{r}$ is oriented orthogonal to that line ($\vec{r}⊥{\vec{m}}_{b}$). Because of these two definitions, the bone line *l*_*b*_(*h*) is fully defined by the tuple (*r*, *φ*) where *r* is in pixel and *φ* in radiant. These parameters span a constrained parameter space of possible lines.

$$\begin{array}{cccc} -\infty < & r<\infty & 0\leq & \varphi \leq \pi \end{array}$$ (20)

This *Hough*-line parameter space was transformed into a constrained and discretized *Hough*-line search space which is described by

$$\begin{array}{c} \begin{array}{cc} r & \in \{r_{k}\;|\;r_{k}=x_{l}+\Delta r\cdot k,\;r_{k}\leq x_{h},\;k\in N_{0}\}\\ \text{with:}\;x_{l} & \equiv -(w_{c}+h_{c})\\ x_{h} & \equiv -x_{l} \end{array} \end{array}$$ (21)

and

$$\begin{array}{c} \varphi \in \{\varphi_{k}\;|\;\varphi_{k}=\Delta \varphi \cdot k,\;r_{k}\leq \pi ,\;k\in N_{0}\} \end{array}$$ (22)

where Δ*r* = 1 and $\Delta \varphi =\frac{\pi }{180}$ are the search resolution parameters.

For each edge pixel in a frame all lines containing this pixel in the discretized parameter space were considered and the counter of the corresponding parameter tuple was increased. After all edge points had been processed, those parameter tuples with counter values larger than a threshold (Threshold_Hough_ = 40) were considered to be bone line candidates. These candidates were sorted by their counter values. The candidates with the 10 highest counter values were selected for further processing (see Fig 9A). The parameters used for the *OpenCV* HougLines function were shown in S3 Table in the S3 Table. If a function parameter is not listed, the default value was chosen. The selected *Hough*-lines were used to calculate a mean bone line. As some structures in the muscle tissue also resulted in detected lines, a clustering algorithm (*DBSCAN* from *SciPy* [36]) was applied to filter for bone lines, as shown in Fig 9B. By means of majority voting, the biggest cluster was chosen and its mean parameter vector was used to describe the bone line. In subsequent steps, only the orientation of the bone line was used. Before clustering, the *Hough*-line parameters were normalized on the expected range for a bone line.

$$\begin{array}{c} {\bar{r}}_{h}\left(n,m\right)=\frac{r_{h}\left(n,m\right)}{\sqrt{w_{c}^{2}+h_{c}^{2}}}\;{\bar{\varphi }}_{h}\left(n,m\right)=\frac{\varphi (n,m)\cdot 2}{\pi } \end{array}$$ (23)

This way distances between parameters in ${\bar{r}}_{h}$ and ${\bar{\varphi }}_{h}$ can be treated equally, which is beneficial for clustering. The function parameter *m* ∈ {0, 1, …, 9} is the index of a bone line parameter set in frame *n*. After this normalization, *DBSCAN* is applied which is a density based clustering algorithm defined by [37]. There are 3 types of data points. 1. a *core point*, which has at least *n*_min,s_ − 1 samples within the distance *ϵ*_core_. 2. a *border point*, which is within the distance *ϵ*_core_ of a *core point*. 3. *noise points* / *outlier*. The latter are not reachable from any point. A point is called reachable, if there is a chain of core points within the distance *ϵ*_core_. Points, which reach each other, are part of the same cluster. For bone line clustering, the amount of core points was set to *n*_min,s_ = 2 and *ϵ*_core_ = 0.02 was chosen. As distance function, the *Euclidean*-distance *L*_2_(•) = ‖•‖_2_ was used. In S4 Table the parameters are listed with their names as given in the SciPy module [36]. Default values were used for those function parameters that are not listed. The bone line was then calculated as the mean of the biggest cluster (*r*_*b*_(*n*), *φ*_*b*_(*n*)) where *n* is the index of the corresponding frame. Based on this findings, the bone line orientation angle relative to the abscissa was calculated as follows.

$$\begin{array}{c} \varphi_{b}(n)-{\hat{\gamma }}_{b}(n)=\frac{\pi }{2}\;\Leftrightarrow \;{\hat{\gamma }}_{b}(n)=\varphi_{b}(n)-\frac{\pi }{2} \end{array}$$ (24)

A derivation of this relationship is shown in Fig 6B. In the used coordinate system, a clock wise rotation is positive. This description of ${\hat{\gamma }}_{b}$ can be used instead of the manual labeled bone orientation ${\tilde{\gamma }}_{b}$.

#### Tracking of the bone reference point via Lucas-Kanade optic flow estimation

As compared to the myotendinous junction, the visual texture of bones in B-mode ultrasound images does not provide distinct visual features consistent for all subjects (Fig 4). Hence, the automated inference of reference points using a deep neural network approach based on transfer learning—such as described in the section *Detection of myotendinous junction using deep neural networks*—is not feasible, as such an approach would require supervised training based on individual features of the bone surface for corresponding subsets of ultrasound images. Instead, for the automated tracking of the bone reference point ${\hat{\vec{p}}}_{\text{b}}$ on the *humerus* from sequences of ultrasound images, an optic flow based approach was used. *Lucas-Kanade* optic flow estimation [38] is based on the assumption that the distribution of apparent velocities induced by motion is constant in a local neighbourhood of an image pixel under consideration. This allows for the temporal tracking of visual features in a sequence of images which do not share distinct textural properties across a set of image sequences. Recent studies have used the *Lucas-Kanade* based estimation of optic flow to temporally track visual features in ultrasound images [26, 27].

To obtain the bone reference point ${\hat{\vec{p}}}_{\text{b}}$ from the data set of B-mode ultrasound images, a region of interest (ROI) representing the bone structure of the *humerus* was computed for each frame of the data set of ultrasound images. The ROI was defined as the area below the visual line separating muscle from bone tissue within the cropped image frames (see paragraph above; Fig 9A). Within the ROI, visual features were selected for the first frame of each ultrasound image sequence using *Shi-Tomasi* corner detection [39]; implemented using *OpenCV* [35]. The maximum number of corners returned via the corner detector was set to *N*_*c*,max_ = 1000 while corners with a quality level of *Q*_*c*_ < 0.25 and a *Euclidean* distance of *d*_*c*,max_ < 3 image pixels were rejected. The size of an average block for computing a derivative covariance matrix over each pixel neighborhood was set to *s*_*b*_ = 3. This resulted in a mean of ${\bar{N}}_{c}=17.34$ corners (standard deviation *σ*_*c*_ = 24.48) detected per analyzed image frame. Subsequently, the detected visual features (or corners, respectively) in the first frame of an image sequence were iteratively tracked in the remaining frames of the corresponding image sequence using a pyramidal implementation of the *Lucas-Kanade* feature tracking algorithm [40]; implemented using *OpenCV* [35]. The maximum number of pyramidal levels taken into account was set to *N*_*p*,max_ = 4 and the size of the search window at each pyramid level to ${\vec{s}}_{w}={\left[20,20\right]}^{\text{T}}$. The termination criteria of the iterative search algorithm were set to *ϵ*_*p*_ = 0.03 for the desired change in parameters at which the algorithm stops and *i*_*p*,max_ = 30 for the maximum number of iterations to compute. The bone reference point ${\hat{\vec{p}}}_{\text{b}}$ was subsequently obtained for each frame *n* in each sequence of ultrasound images via computation of the centroid (i.e. the mean) of the visual features with coordinates [*f*_*x*_, *f*_*y*_]^*T*^ tracked via *Lucas-Kanade* optic flow estimation according to

$$\begin{array}{c} {\hat{\vec{p}}}_{\text{b}}\left(n\right)={\left[\bar{f_{x}}\left(n\right),\bar{f_{y}}\left(n\right)\right]}^{T} \end{array}$$ (25)

whereas features leaving the ROI during tracking within an image sequence due to movement of the bone in relation to the ultrasound sensor were discarded.

#### Comparison of manual and automatic feature extraction

The automatic feature extraction was compared to the manually extracted data per feature.

When evaluating the tracking of the myotendinous junction, the absolute movement between states (unloaded, loaded) was compared,

$$\begin{array}{c} ∥\Delta {\hat{\vec{p}}}_{mt}{∥}_{2}-{∥\Delta {\tilde{\vec{p}}}_{mt}∥}_{2} \end{array}$$ (26)

as the investigator in the manual method and the automatic method did not necessarily track the same feature of the myotendinous junction. Tracking of the bone line was evaluated separately for the loaded and unloaded state as the angle of the bone line was comparable on a frame basis.

$$\begin{array}{c} {\hat{\gamma }}_{b}(n_{u})-{\tilde{\gamma }}_{b}(n_{u}),\;\;{\hat{\gamma }}_{b}(n_{l})-{\tilde{\gamma }}_{b}(n_{l}) \end{array}$$ (27)

In case of the bone reference point the absolute movement of a manually chosen feature on the bone (see Fig 5) and the absolute movement of an automatically chosen feature by *Lucas-Kanade* was compared. The comparison of the absolute distance between steady states

$$\begin{array}{c} ∥\Delta {\hat{\vec{p}}}_{b}{∥}_{2}-{∥\Delta {\tilde{\vec{p}}}_{b}∥}_{2} \end{array}$$ (28)

The results of these comparisons are shown in Fig 11. For each comparison a mean absolute error (MAE) and the *Pearson* correlation coefficient (R) is calculated.

#### Calculation of myotendinous junction displacement

The myotendinous junction displacement parallel to the bone needed to be calculated analog to the manual data extraction. For that, Eqs (14) and (17) still held true. For each manually extracted feature (${\tilde{\vec{p}}}_{\text{mt}}$, ${\tilde{\vec{p}}}_{b}$, ${\tilde{\gamma }}_{b}$) there is an automatically extracted counterpart (${\hat{\vec{p}}}_{\text{mt}}$, ${\hat{\vec{p}}}_{b}$, ${\hat{\gamma }}_{b}$). These features are compared in Fig 11. It appears that the error between manually and automatically labeled absolute bone reference point movement (see Eq (8)) correlates with the wrist force *F*_*w*_ (*R* = −0.58). This finding is later discussed. Therefore, for further steps the bone reference point was used from the manually labeled data set ${\tilde{\vec{p}}}_{b}\left(n\right)$ instead. Consequently, the myotendinous junction displacement vector is defined as follows.

$$\begin{array}{c} \begin{array}{c} {\hat{\vec{p}}}_{\text{b,mt}}\left(n_{k}\right)=R^{-1}({\hat{\gamma }}_{b}\left(n_{k}\right))\cdot ({\hat{\vec{p}}}_{\text{mt}}\left(n_{k}\right)-{\tilde{\vec{p}}}_{\text{b}}\left(n_{k}\right))\\ \text{with}\;k\in \{\text{u},\text{l}\} \end{array} \end{array}$$ (29)

$$\begin{array}{c} \Delta {\hat{\vec{p}}}_{\text{b,mt}}(\Theta ,F_{\text{w}})={\hat{\vec{p}}}_{\text{b,mt}}\left(n_{\text{l}};\Theta ,F_{\text{w}}\right)-{\hat{\vec{p}}}_{\text{b,mt}}\left(n_{\text{u}};\Theta ,F_{\text{w}}\right) \end{array}$$ (30)

Then the displacement parallel to the bone is defined as:

$$\begin{array}{c} \Delta {\hat{L}}_{\text{mt}}(\Theta ,F_{\text{w}})={\left[\Delta {\hat{\vec{p}}}_{\text{b,mt}}(\Theta ,F_{\text{w}})\right]}_{x} \end{array}$$ (31)

For the steady states *n*_*u*_ and *n*_*l*_, the same frames were picked as for the manual data extraction. Data for $\Delta {\hat{L}}_{\text{mt}}$ is shown in Fig 7B for subject 0, as example.

### Measurement of elbow joint torque

For the prediction of the myotendinous junction movement, the tendon force needs to be calculated. During the experiment, the wrist force *F*_*w*_ was measured. From this force, the net elbow torque was calculated for the unloaded and loaded state. Later in this work, this data was used to estimate the torque which is generated by the *biceps brachii* from which the tendon force can be estimated.

#### Wrist force extraction

The unfiltered wrist force $F_{\text{w}}^{\text{raw}}$ and data from B-mode ultrasound frames were not synchronized. However, steady states can be identified in $F_{\text{w}}^{raw}$. Since it was unknown at which time during the steady state the frames were captured, the mean of the wrist force in each steady state was calculated. The force contributing to a moment at the elbow joint during the unloaded state was assumed to be zero. A measured force during this time window therefore had to be attributed to the preloading of the wrist strap. The measured mean force during the loaded state was therefore offset by the mean force during the unloaded state in order to obtain the actual elbow moment generating force *F*_w_.

$$\begin{array}{c} F_{\text{w}}=\frac{\sum_{i=n_{\text{l0}}}^{n_{\text{l1}}}F_{\text{w}}^{\text{raw}}\left(i\right)}{n_{\text{l1}}-n_{\text{l0}}}-\frac{\sum_{i=n_{\text{u0}}}^{n_{\text{u1}}}F_{\text{w}}^{\text{raw}}\left(i\right)}{n_{\text{u1}}-n_{\text{u0}}} \end{array}$$ (32)

Where *n*_l0_ is the first sample of the loaded state and *n*_l1_ is the last sample of the loaded state. The same principle applies to the unloaded state (*n*_u0_ first sample—*n*_u1_ last sample).

#### Torque at the elbow joint

The moment arm *l*_w_(Θ) changes over the elbow angle due to the construction of the support rack (Fig 3). For this reason, the distance between the elbow joint and the wrist strap was measured for each elbow angle Θ. With this measurement, the corresponding elbow joint torque *τ*_e_ was calculated.

$$\begin{array}{c} \tau_{\text{e}}(\Theta ,F_{\text{w}})=l_{\text{w}}\left(\Theta \right)\cdot F_{\text{w}} \end{array}$$ (33)

This elbow torque was later used to estimate the torque which was generated by the *biceps brachii*.

### Modeling the tendon and parameter fitting

As described in the section *Mammalian tendon modeling*, there are two major tendon models. In this work, the model $F_{T}^{\text{Rack}}(l_{T}-l_{0,T};\vec{\rho })$ was used. It is derived from elastic properties as found in experiments measuring the stiffness of a mammalian tendon *in vitro*. As the experiment in this work was conducted *in vivo*, the variables *l*_0,*T*_,*l*_*T*_, $\vec{\rho }$, *F*_*T*_ were not known a priory.

The absolute tendon length *l*_*T*_ and the resting tendon length *l*_0,*T*_ remained unknown, whereas the tendon force *F*_*T*_ and the stiffness parameter vector $\vec{\rho }$ have been estimated. The tendon force was estimated by means of a two dimensional tendon path model. The stiffness parameter vector was estimated by fitting the tendon model on the measurements of the myotendinous junction displacement. Instead of the absolute tendon length, the change in length from rest to the current state was used Δ*l*_*T*_ = *l*_*T*_ − *l*_0,*T*_. It was estimated based on the myotendinous displacement parallel to the bone (as observed in the B-mode videos) and by the assumption:

$$\begin{array}{c} \Delta l_{T}\approx \Delta L_{mt} \end{array}$$ (34)

**Fig 10:**
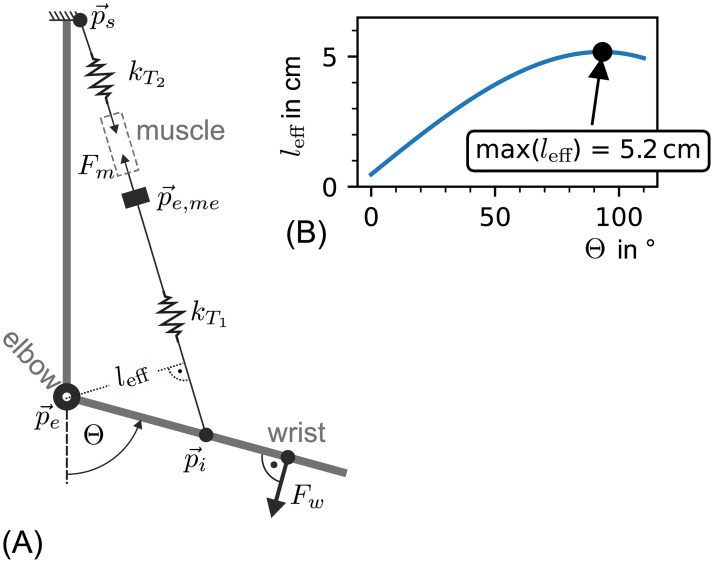
(A) Sketch of the implemented elbow moment arm model with (B) example data of subject 0. (A) Schematic depiction of the applied biomechanical model of muscle and tendon in the elbow joint. *k*_*T*1_ and *k*_*T*2_ are non linear spring elements representing the lower and upper *biceps brachii* tendons. The shoulder and lower arm insertions are also marked (${\vec{p}}_{s}$ and ${\vec{p}}_{i}$). These points were determined based on anatomic landmarks as formulated in Eq (37). The elbow joint was abstracted as revolute joint. It was located at ${\vec{p}}_{e}$. The effective lever length of subject 0 is shown in (B) over the elbow joint angle Θ. The maximal effective lever length is marked at (Θ = 93.3^∘^, *l*_eff_ = 5.2cm).

In this study, only the distal *biceps brachii* tendon was observed with ultrasonography. The force exerted on the distal tendon $F_{T_{1}}$ was estimated based on the measured elbow joint torque *τ*_*e*_ as given in Eq (33) and a model of the abstracted tendon path (cmp. Fig 10A). The tendon path model was parameterized by measurements of anatomic landmarks as given in Eq (37).

The parameter vector $\vec{\rho }$ was estimated with a fit on the collected subjects’ data. As a target function for the fit, the myotendinous displacement Δ*L*_*mt*_(*F*_w_) as a function of the wrist force was used. This is because the wrist forces *F*_w_ were voluntarily adjusted by the subjects during the experiments and have only a small variance per force interval (see Fig 7). For the myotendinous displacement Δ*L*_*mt*_(*F*_w_), the formulation for $\Delta l_{T}^{\text{Rack}}(F_{T},\vec{\rho })$ from Eq (12) was used.

#### Estimation of the tendon force

In this work, tendon displacement was predicted based on the measured wrist force *F*_w_ and an abstraction of the skeletal geometry as shown in Fig 10A. The model results in an effective lever *l*_eff_. From this, the tendon force could be calculated by

$$\begin{array}{c} F_{T1}\left(F_{\text{w}}\right)=\frac{\tau_{\text{bic}}(\tau_{\text{e}})}{l_{\text{eff}}} \end{array}$$ (35)

since the elbow joint was assumed to be a revolute joint. The torque *τ*_bic_ is generated by the *biceps brachii*. It was estimated based on the elbow joint torque, as it was not directly measured.

$$\begin{array}{c} \tau_{\text{bic}}(\tau_{\text{e}})=\tau_{\text{e}}-\tau_{\text{r}} \end{array}$$ (36)

In this equation, *τ*_r_ is the torque generated from other sources (i.e. friction, other muscle groups, surrounding tissue). The forearm, which contains two bones (*ulna* and *radius*) as mechanical base structure, was abstracted as a single lever. The distal *biceps brachii* tendon *T*_1_ inserts into this lever at ${\vec{p}}_{i}$ with the same distance *l*_i_. As distance *l*_i_ the distance between the elbow and the insertion into the *radius* was used. The *fascial* insertion of the distal *biceps brachii* *aponeurosis* was ignored.

The two headed *biceps brachii* was abstracted to a single head inserting at ${\vec{p}}_{s}$ into the shoulder. With the coordinate systems origin placed within the elbow revolute joint, the points

$$\begin{array}{cccccc} {\vec{p}}_{i} & =R(\Theta )[\begin{array}{c} 0\\ -l_{\text{i}}(l_{u})\\ 0 \end{array}] & {\vec{p}}_{s} & =[\begin{array}{c} l_{\text{cphp}}\\ l_{a,e}\\ 0 \end{array}] & {\vec{p}}_{e} & =[\begin{array}{c} 0\\ 0\\ 0 \end{array}] \end{array}$$ (37)

were defined (see Fig 10A). Here, the distance from the distal tendon insertion point to the elbow joint *l*_i_ was assumed to be proportional to the length of the *ulna* (*l*_*i*_ ∝ *l*_*u*_).

$$\begin{array}{c} l_{i}\approx 0.186\cdot l_{u} \end{array}$$ (38)

The conversion factor was set based on the proportions depicted in figure. 334 of [41]. The effective lever length can be calculated as follows.

$$\begin{array}{c} \left[\begin{array}{c} 0\\ 0\\ l_{\text{eff}} \end{array}]=[{\vec{p}}_{i}(\Theta )-{\vec{p}}_{e}]\times \vec{e}({\vec{p}}_{s},{\vec{p}}_{i}(\Theta )\right) \end{array}$$ (39)

Where $\vec{e}(\vec{x},\vec{y})=\frac{\vec{x}-\vec{y}}{{∥\vec{x}-\vec{y}∥}_{2}}$ is a unit vector in the direction of $\vec{x}-\vec{y}$. The course of the effective lever length *l*_eff_ over the elbow angle for subject 0 is shown exemplarily in Fig 10B.

With Eqs (35) and (39) the estimation of tendon force is complete. It was used for fitting the tendon model $\Delta l_{T_{1}}$ onto the extracted myotendinous junction movement $\Delta {\tilde{L}}_{\text{mt}}$ and the wrist force *F*_w_.

#### Fitting the tendon model to data

Due to the small amount of data per elbow angle and its high variance in the myotendinous displacement Δ*L*_mt_, it was preferable to fit the model onto all angles in one process instead of breaking it down into several sub-processes—one for each angle. The material properties of the tendon were assumed invariant, also in terms of changes of the elbow angle. However, if the predicted biceps torque is less than the elbow torque (*τ*_*r*_ > 0, see Eq (36)) the tendon parameters are effected. This means that, when *τ*_*r*_ depends on the elbow angle, it seems as if the tendon parameters are changing depending on the angle. The same holds true for the effective moment arm *l*_eff_. These effects are further analyzed in the *results and evaluation* section. Therefore, it was assumed that only a subset (or sub-range) of the measured elbow angles can be explained by the current model. Within this sub-range, *τ*_*r*_ ≈ 0 was assumed. Additionally, *l*_eff_ was assumed to predict the effective lever length for that sub-range. As the valid elbow angle range was not known a priori, it was narrowed down in an optimization process. In this optimization process, fits of the model for decreasing ranges of Θ were performed. Later, the elbow angle range was used as a fit result whose mean cost per sub-range is stable as compared to all angle ranges (cmp. Figs 12A and 13A, example data from three subjects). For each such elbow angle range fit, the error function

$$\begin{array}{c} \begin{array}{cc} & g(\vec{\rho };\Theta_{\text{lb}},\mu (•))\\ = & \sum_{k\in S_{\Theta_{\text{lb}}}}\mu {\left(\Delta L_{mt}(\Theta \left[k\right],F_{T1}\left(F_{\text{w}}\left[k\right]\right);\vec{\rho })-\Delta {\tilde{L}}_{\text{mt}}\left[k\right]\right)}^{2} \end{array} \end{array}$$ (40)

was minimized. The error function *g* calculates a sum of differences between the predicted and measured tendon displacement. The function *μ*(•) was used to distort the error function to cope with variance in the data. In this case, *μ*(•) = ln(•+ 1) was chosen. The functions Θ[*k*], *F*_w_[*k*] and $\Delta {\tilde{L}}_{\text{mt}}\left[k\right]$ are discrete functions returning the respective values of a data point indexed by *k*. The set $S_{\Theta_{\text{lb}}}$ (lb for lower bound) contains all indices of data points where Θ_lb_ ≤ Θ. Therefore, it is possible to calculate fits over subsets of elbow angles.

$$\begin{array}{c} \begin{array}{cc} \underset{\vec{\rho }}{min} & \;F(\vec{\rho })=0.5\cdot g\left(\vec{\rho };\Theta_{\text{lb}},\text{ln}(•+1)\right)\\ \text{with} & \;{\vec{\rho }}_{\text{lb}}\leq \vec{\rho }\leq {\vec{\rho }}_{\text{ub}} \end{array} \end{array}$$ (41)

This minimization was done by performing fits with

$$\begin{array}{c} \Theta_{\text{lb}}\in S_{\Theta_{\text{lb}}}\equiv \{0{\;}^{\circ },15{\;}^{\circ },30{\;}^{\circ },45{\;}^{\circ },60{\;}^{\circ },75{\;}^{\circ }\} \end{array}$$ (42)

as lower boundaries for the elbow angle subsets which resulted in parameter sets ${\vec{\rho }}_{\Theta_{\text{lb}}}$ for each value of Θ_lb_. The mean cost per subset was calculated as follows.

$$\begin{array}{c} \epsilon \left(\Theta_{\text{lb}}\right)=\frac{g(\rho_{\Theta_{\text{lb}}},\Theta_{\text{lb}})}{\left|n_{\Theta_{\text{lb}}}\right|} \end{array}$$ (43)

Where $n_{\Theta_{\text{lb}}}$ is the amount of data-points of the corresponding angle subset defined by Θ_lb_. As an optimization algorithm, the non linear function optimizer *trust region reflective* from the Python *SciPy* package [36] was used. For the optimization process

$$\begin{array}{c} {\vec{\rho }}_{0}=[\begin{array}{c} 0.1\\ 0.1\\ 0.002 \end{array}] \end{array}$$ (44)

was used as the initial parameter vector. As lower and upper boundaries

$$\begin{array}{cccc} {\vec{\rho }}_{\text{lb}} & =[\begin{array}{c} {\text{eps}}_{64}\\ {\text{eps}}_{64}\\ {\text{eps}}_{64} \end{array}], & {\vec{\rho }}_{\text{ub}} & =[\begin{array}{c} 1e6\\ 1e6\\ 0.035 \end{array}] \end{array}$$ (45)

were used. Here, eps_64_ is the machine epsilon of the 64 bit float datatype. Results of the fitting process are depicted in Figs 12 and 13 and evaluated in the following section.

## Results

In this work, a manual and a (semi-) automatic method of extracting data (myotendinous junction displacement in a bone local coordinate system) from B-mode ultrasound images were used. Both methods where evaluated based on the displacement of the distal *biceps brachii* tendon during the generation of different wrist forces at different elbow angles. Subsequently, tendon models were fitted to the manual and (semi-) automatically extracted data from three subjects (cmp. Figs 12 and 13). Special emphasis was placed on fitting the models not only to one elbow angle but to subsets from the entire range of elbow angles.

### Comparison of manual and (semi-) automatically extracted data from B-mode ultrasound videos

**Fig 11:**
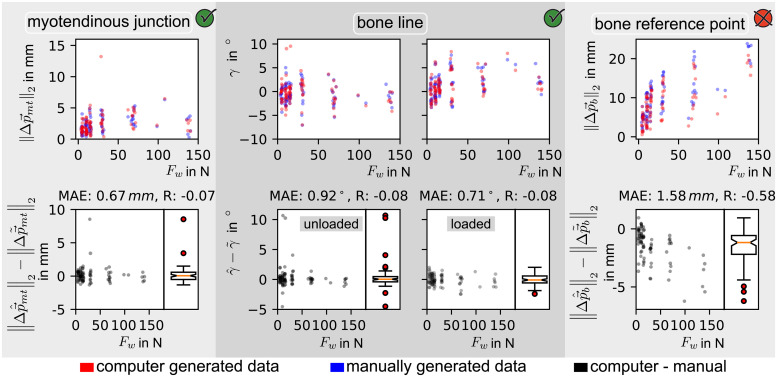
Comparison for each feature between manual and automatic data extraction. The green check mark in the upper right corner indicates that the corresponding method was used for evaluation in this work. The red cross indicates that the manually labeled data was preferred over the automatically extracted data.

The myotendinous displacement parallel to the bone was either extracted manually or automatically from frames of B-mode ultrasound videos. The two methods are compared for subject 0 in Figs 7 and 11. During automatic feature extraction, for each feature (*myotendinous junction*, *bone line* and *bone reference point*) a distinct algorithm was applied (Fig 8: left, middle and right, respectively). Thus, each of the three algorithms needed to be evaluated separately. For each feature tracking the result was compared with the manual tracking as ground truth. The difference from manual to automatic evaluation is shown over the wrist force in Figs 7C and 11.

#### Tracking of the myotendinous junction

The comparison of the absolute myotendinous junction displacement between manual and automatic data extraction resulted in a mean absolute error (MAE) of 0.67 mm ≐ 9:0 pixel. The differences were distributed approximately symmetrical around zero, with two outliers. As for the outliers, the neural network extracted a larger movement than the manual evaluation. The difference of absolute movements correlates with the wrist force by R = −0.07 (see Fig 11, left), where R is the *Pearson* correlation coefficient.

#### Tracking of the bone line

Tracking of the bone line was evaluated separately for the loaded and unloaded state. In both states, the computation of the mean absolute error resulted in MAE < 1^∘^ (see Fig 11, middle), which was below the resolution of the *Hough*-line transformation. Apart from outliers, the mean absolute errors for both states were distributed around zero. This is due to image cropping, which mostly affected the unloaded state due to a smaller distance between bone and ultrasonic probe. Using a higher cropping window would have led to false bone line detection within muscle tissue. In either, the loaded and the unloaded state, the bone line angle *γ* correlated with the wrist force *F*_*w*_ by R = −0.08 (*Pearson* correlation coefficient).

#### Tracking of the bone reference point

The comparison of the absolute distance between steady states correlated with *F*_*w*_ by R = −0.58 (*Pearson* correlation coefficient). This resulted in an error distribution around an offset value unequal to zero.

Comparing the (semi-) automatic data extraction with the manual data extraction for subject 0 led to a MAE of 1.15mm (see Fig 7C). Therefore, with a mean absolute error difference close to one millimeter, the results of both methods were assumed to be equivalent.

### Results of the tendon model fitting process

**Fig 12:**
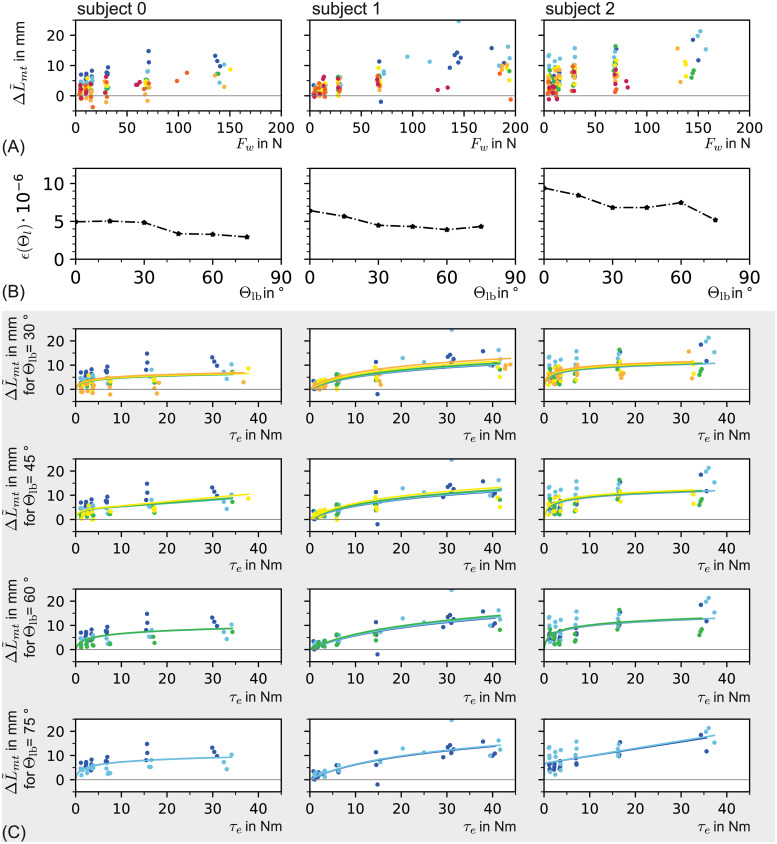
Manually extracted data and corresponding fits for three subjects in column-wise arrangement. **(A)** ***Myotendinous junction*** **displacement** $\Delta {\tilde{L}}_{mt}$ as manually extracted from B-Mode videos plotted over the wrist force *F*_*w*_. **(B) Normalized costs of each angular subset fit** (see Eq (43)). **(C) Fits of the angular subsets**Θ_*lb*_ = 30^∘^, Θ_*lb*_ = 45^∘^,Θ_*lb*_ = 60^∘^,Θ_*lb*_ = 75^∘^. (Same color coding as in Fig 7).

**Fig 13:**
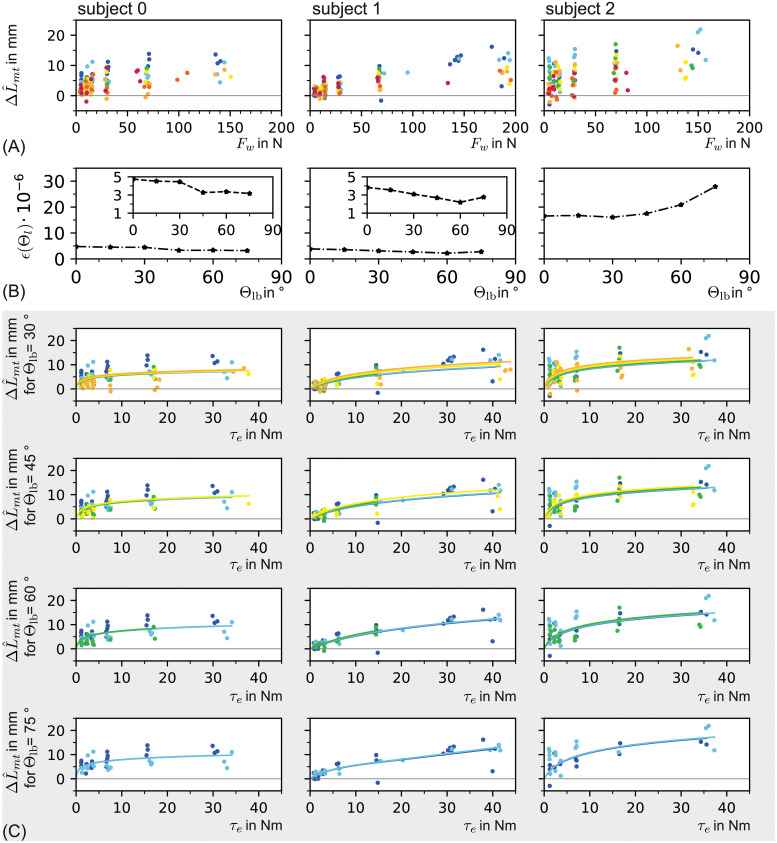
Fits on (semi-) automatically extracted data for three subjects in column-wise arrangement. (A) *Myotendinous junction* displacement $\Delta {\hat{L}}_{mt}$ which is (semi-) automatically extracted from B-Mode videos plotted over the wrist force *F*_*w*_. (B) Normalized cost of each angular subset fit (see Eq (43)). (C) Fits of the angular subsetsΘ_*lb*_ = 30^∘^, Θ_*lb*_ = 45^∘^,Θ_*lb*_ = 60^∘^,Θ_*lb*_ = 75^∘^. (Same color coding as in Fig 7).

As described in the *Modeling the tendon and parameter fitting* section, elbow angle sub-ranges of the manually and (semi-) automatically extracted data were used to fit the tendon model for three subjects as examples. The fitting results of the manual and (semi-) automatically extracted data are shown in Figs 12 and 13, respectively.

In case of the manually extracted data, the fits over elbow angle sub-ranges showed that there are sub-ranges with nearly identical mean cost *ϵ* (cmp. Fig 12B). For smaller Θ_lb_ (larger angle sub-range), the mean costs increased for all three subjects.

In case of automatically extracted data, this trend only held for subject 0 (cmp. Fig 13B).

For subject 0 and 1 the optimal elbow angle sub-range was identical for $\Delta {\hat{L}}_{mt}$ (manual) and $\Delta {\tilde{L}}_{mt}$ (automatic). For subject 2 the optimal subset deviated between manual (Θ_lb_ = 75) and (semi-) automatically extracted data (Θ_lb_ = 30).

It was common for all fits that with decreasing angle, a higher tendon displacement was predicted. In contrast, the measured tendon displacement generally decreased for each elbow torque if the elbow angle became smaller. Thus, the model had a tendency to overestimate the myotendinous junction movement for decreasing elbow angles.

**Fig 14:**
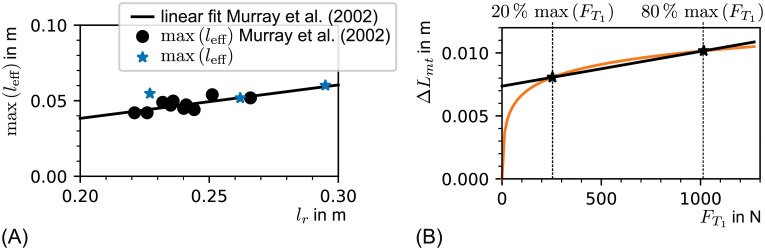
**(A) Validation of the maximal effective lever length.** The maximal effective lever length *l*_eff_ over the *radius* length is shown as black dots, as presented in the cadaver study by Murray et al. [42]. The linear fit of Murray’s data is shown as black line. The blue stars are the maximal effective lever lengths of the subjects in this study. A complete course of the effective lever length is shown in Fig 10B for subject 0. **(B) Non linear and linear prediction of myotendinous junction movement.** The non linear prediction of the myotendinous junction movement over tendon force is shown in orange. The prediction is based on Eq (10). As parameter set, the resulting fit on (semi-) automatically extracted data from all angels Θ ≥ 75^∘^ of subject 0 was used. In addition, a linear model is shown in black. This linear model was parameterized such that the linear function includes the predicted values for 20% and 80% of the maximal tendon force generated during voluntary contraction $\left(\text{max}\left(F_{T_{1}}\right)=1270\;\text{N}\right)$. The stiffness of the depicted linear model was *c*_lin,20-80_ = 362N/mm.

In order to compare these fitting results with the findings in [9] a linear stiffness was calculated for each fit on the angle subranges Θ ≥ 30 (see Table 1). The linear stiffness was computed analog to [9] with the tendon lengthening at 20%Â max(*F*_*T*1_) to 80% max(*F*_*T*1_). An example linear stiffness is shown in Fig 14B in comparison to the nonlinear tendon stiffness function.

**Table 1:** For a selection of elbow angle sub-ranges, the linear stiffness constant between tendon lengthening at 20% max(*F*_*T*1_) to 80% max(*F*_*T*1_) is shown for (semi-) automatically extracted data (auto) and the manually extracted data (man.). In addition, the optimal angle sub-range is highlighted in gray for manually and automatically extracted tendon displacement (cmp. Figs 12B and 13B).

experiment		*c*_lin,20−80_ in $\frac{\text{N}}{\text{mm}}$			
subj.	data	Θ ≥ 30	Θ ≥ 45	Θ ≥ 60	Θ ≥ 75
0	auto	444	318	312	362
	man.	462	166	318	338
1	auto	209	170	127	103
	man.	178	151	122	117
2	auto	211	185	145	113
	man.	342	284	248	59

## Discussion

In the work described here the displacement of the *biceps brachii* myotendinous junction parallel to the *humerus* was extracted from B-mode ultrasound images as part of an *experimental setup* that also allowed determination of applied loading forces and elbow joint angles. A manual and an automatic *method to extract the relative motion of the myotendinous junction* based on visual features from ultrasound images were developed. The performance of both methods was compared. Using experimental data from three subjects, a tendon model was fitted to the data of both methods used for extracting the myotendinous junction displacement. In both cases a reliable fit was achieved.

### Experimental setup

In the experiment, the myotendinous junction was observed with a linear ultrasonic probe array, while the wrist force was measured asynchronously with respect to the ultrasonic measurement (cmp. Fig 3). As a consequence, the collected data and the resulting fits could only be used for the examination of steady states of the tendon behaviour. Any dynamic processes such as creepage were not taken into account.

The myotendinous junction was observed in a saggital plane with a linear ultrasonic probe array (cmp. view I in Fig 4B). The moytendinous junction, and also the course of the *aponeurosis*, has a three-dimensional shape and was not oriented in a particularly aligned way in the saggital plane (cmp. Fig 4B). Therefore, during muscle contraction, the motion of the myotendinous junction had components within the saggital imaging plane as well as outside the plane, e.g. perpendicular to it. The latter were not detected in the presented setup.

Additionally, the *biceps* muscle bulges during contraction, changing the outer contour of the arm, which also changed the relative orientation of the sensor probe to the myotendinous junction. As the ultrasonic sensor probe was handheld, the operator attempted to compensate tilting during the execution of the experiment. If the sensor probe tilted too much the bone reference point moved out of the imaging plane and therefore could not be tracked. These cases were excluded from the data set.

A future extension of the setup could include a two-dimensional ultrasonic sensor array, which would support the investigation of a three-dimensional measurement volume. This would allow consistent tracking of the junction and the bone reference point independent of tissue deformation and sensor tilting during contraction. Note the extent of dispersion of the myotendinous junction displacement with the current measurement setup (Figs 12A, 13A). The use of volume data could decrease the error in myotendinous junction displacement measurement.

### Extraction of the myotendinous junction displacement parallel to the *humerus*

From the ultrasonic measurements, three features where extracted manually and with a (semi-) automatic approach. These features were the tracked movements of, first, the myotendinous junction, second, the *humerus* bone surface (bone line), and third, a reference point on the bone surface. The combination of these features allowed a calculation of the myotendinous junction movement in a bone local coordinate system, unlike other works in which the myotendinous junction was tracked in a skin local coordinate system (e.g. [9, 10] used ultrasonic reflective markers on the skin as reference points).

Of these three features, the automatic tracking methods for myotendinous junction and bone orientation via bone line tracking both proved to be feasible. It should be noted, that during preprocessing of the ultrasonic images a Gaussian filter was used, which could potentially contribute to some amount of error in the detection of the bone line and bone reference point. However, the automatic extraction of the bone reference point (represented by “some kind of texture” of the bone surface) based on the *Lucas-Kanade* was not used due to the correlation of the difference between manual and *Lucas-Kanade* tracking with the wrist force (R = −0.58). As the investigator selected distinct reference points (e.g. where *brachialis* fibers connect with the bone), the manual labeling was assumed to be the ground truth. This assumption led to the conclusion that there was a systematic error proportional to the wrist force *F*_*w*_ in case of *Lucas-Kanade* tracking of the bone reference point.

As a consequence, the tracking of the bone reference point, as part of the procedure to estimate $\Delta {\hat{L}}_{mt}$, was performed manually according to Eq (31). Due to this one manual step, the former automatic method was regarded as (semi-) automatic.

In this study the myotendionous junction is observed at the point where the aponeurosis forks into the muscle (here described as myotendionous junction). The movement of the myotendionous junction is than solely attributed to changes in tendon length during model building. Further work could include more complex model configurations of the myotendinous junction such as the aponeurosis and fascia.

As mentioned before, the usage of a handheld linear ultrasonic probe limited the performance of data extraction for the myotendinous junction. It seems plausible, that this limitation also affected the tracking of the bone reference point. The bone was the structure with the greatest distance to the ultrasonic sensor. Therefore, tilting of the sensor probe had a larger effect on bone imaging as compared to the myotendinous junction. Also here, the usage of a two-dimensional ultrasonic sensor array to get volumetric data could be beneficial in future. The fitting of volumetric models of anatomical features could lead to a reliable feature tracking for all three anatomic features (bone orientation, bone reference point and myotendinous junction).

### Tendon model and its fitting

During model building, three major assumptions were made that strongly influenced the observed model behaviour. First, there is no preloading of the tendon. Second, the effective lever length *l*_eff_ is calculated based on a two-dimensional tendon path model as depicted in Fig 10. Third, the resulting elbow torque is solely generated by the *biceps brachii* (*τ*_*r*_ = 0).

#### The effect of *l*_eff_ miscalculation

If the effective lever length of the *biceps brachii* was miscalculated this would lead to an elbow angle dependent over- or underestimation of the tendon force *F*_T1_. A quantitative analysis was not applicable as *l*_eff_ could not be obtained from the participants in the presented measurement setup (see Fig 3). Direct *in vitro* measurements of the effective lever length of the lower *biceps brachii* tendon exist. Murray et al. presented data of the peak effective moment arm of the distal *biceps brachii* tendon over the length of the *radius* [42]. This data is depicted in Fig 14A. In addition, the peak effective moment arm of subject 0 to 2 over their *radius* length *l*_*r*_ was entered into the graph. The data of subject 0 to 2 lay within the trend of [42]. Therefore, it was assumed that the maximal moment arm (between 60^∘^ and 100^∘^, according to [42]) and effective moment arms for angles near the maximum were predicted reasonably well by the presented model.

#### The effect of *τ*_*r*_ on the measurement

During model building, *τ*_*r*_ (as defined in Eq (36)) was assumed to be zero as the participant had been advised to flex the elbow joint with no co-contraction. But during the experiment it turned out that subjects had the tendency to co-contract to reach the desired force levels. If co-contraction was noticed during the experiment, it was pointed out to the subject with the request to stop co-contracting even if the requested force level could not be reached exactly. If other than the assumed muscles for co-contraction were dominant, the actual residual torque would be negative ($\tau_{r}^{\text{actual}}<0$).

$$\begin{array}{c} \tau_{\text{bic}}-\tau_{\text{bic}}^{\text{actual}}=\tau_{r}^{\text{actual}} \end{array}$$ (46)

Due to Eq (46), co-contraction would lead to an underestimate of the torque generated by the *biceps brachii* ($\tau_{\text{bic}}<\tau_{\text{bic}}^{\text{actual}}$) and therefore also an underestimate of Δ*l*_mt_. Analog an overestimate of the torque generated by the *biceps brachii* ($\tau_{\text{bic}}>\tau_{\text{bic}}^{\text{actual}}$) is possible, if additional flexor muscles were engaged during contraction *τ*_r_ > 0. Whenever Δ*L*_mt_ was overestimated this seemed to be the dominant error source. During the fit process shrinking sub-ranges of Θ were fitted and led to increasing optimal fit results (in case of manual data extraction). Shrinking sub-ranges mean that lower angles were excluded from the fit. Considering that the *biceps brachii* has its approximate optimal working point at Θ ≈ 115^∘^ [43], it seemed to be plausible that the *biceps brachii* was not the mainly engaged muscle for small elbow angles (arm extended).

#### The effect of tendon preloading on elbow torque estimation based on wrist force measurement

The preloading of the tendon is not directly measurable *in vivo*. A deceptive way to estimate preloading would be to observe the force at the wrist during the unloaded state. However, even if the only physiological torque source was the *biceps brachii* the preloading would not be distinguishable from preloading of the wrist strap (cmp. Fig 3). If additional torque sources were assumed, the lower tendon of the *biceps brachii* could be preloaded even if the resulting torque sum at the elbow joint would be zero.

Therefor, tendon pre-loading remains to be unknown. If tendon pre-loading occurs, it would lead to a gradual shift towards the linear region (see Fig 2).

#### Discussion of the tendon force estimation

However, during model building it was assumed that the *biceps brachii* was the only active muscle. This assumption might have led to an overestimation of the tendon force and, as a consequence, to an overestimation of the tendon stiffness. Shukla et al. [4] investigated the properties of the *biceps brachii* tendon *in vitro*. They found a mean failure load force of the tendon-*radius* insertion at 90^∘^ elbow flexion of 360N (SE 120N). In this study, a mean tendon force during maximal voluntary contraction at 90^∘^ elbow flexion of $F_{T_{1}}=1388\;\text{N}$ (SE 84N) was found. This force seems to be too high considering the failure load found by Shukla et al. [4]. However, the comparison with Shukla et al. is difficult because the tearing of the tendon from the bone was measured *in vitro*. *In vivo*, the distal *biceps brachii* tendon not only inserts into the bone but also into the forearm fasciae, which leads to a presumably higher failure force. In addition, other flexor muscles, such as *brachialis* and *radio brachialis*, may also contribute to an overestimation of the tendon force. However, the actual force generated by the *biceps brachii* could be estimated by using the ratio of the maximal cross sectional area of the *biceps brachii* and the combined maximal area of all elbow flexor muscles. This is based on the assumption that the cross sectional area of a muscle is directly proportional to the force generated during maximal voluntary contraction. According to Erskine et al. [44], the *biceps brachii* makes up 41.8% (SE 1.2%) of the overall maximal cross sectional areas of all flexor muscles. Following this line of thoughts, the *biceps brachiii* would exert a force of 580N (SE 50N). If it is assumed that this force was distributed to the bone and fascial insertions, a reasonable maximal voluntary force is reached which is comparable to the findings of Shukla et al.. Without such an estimation of the force generated by the *biceps brachii*, the tendon force is overestimated. An overestimate of the tendon force leads to an overestimate of the tendon stiffness.

In the captured ultrasonic videos it was noticeable that the *brachialis* muscle group changes its diameter. This, however, speaks in favour of an increasingly negative *τ*_*r*_ for smaller elbow angles. Therefore, the parameterized tendon model was not used to determine tendon stiffness (change in tendon force per change in tendon length). Rather, the presented model allowed the tendon length change to be determined based on the elbow torque. Further work should focus on the contribution of the remaining flexor muscles on the resulting elbow torque to predict *τ*_*r*_ more accurately.

Smart et al. have conducted a comparable study. In that study, the measured elbow torque was also attributed only to the contraction of the *biceps brachii* [9]. Their results should therefore be comparable to those in this work. They defined the tendon stiffness as the slope of a linear displacement over force function. This function was defined to include the tendon displacement at 20% and 80% of maximum voluntary contraction. For comparison, the same linear stiffness was calculated for the fit results of this study as demonstrated in Fig 14B and listed in 1. Smart et al. found a stiffness of $c_{\text{lin,}}\;{20-80}^{\text{young}}=170.1\pm 132.9\frac{\text{N}}{\text{mm}}$ for 9 young participants [9]. In the present study, for the optimal fit of each subject’s elbow angle subset (Table 1) a mean stiffness of ${\hat{c}}_{\text{lin,}}\;20-80=233.3\;\frac{\text{N}}{\text{mm}}$ ($\text{SE}\;56.1\;\frac{\text{N}}{\text{mm}}$) (in case of the automatic data extraction) and ${\tilde{c}}_{\text{lin,}}\;20-80=173.0\;\frac{\text{N}}{\text{mm}}$ ($\text{SE}\;69.0\;\frac{\text{N}}{\text{mm}}$) (for the manually extracted data) was found. These linear stiffness results are considered not to be significantly different (two-sided T-test) as compared to the findings of Smart et al. [9] ($\hat{p}=0.47$ for the automatically extracted data, and $\tilde{p}=0.97$ for the manually extracted data). A general drawback of the linear stiffness even as medical parameter is, that small variances in 20% of $\text{max}(F_{T_{1}})$ can lead to large variances in *c*_lin,20-80_ due to the non-linearity in the toe region (cmp. Fig 14B).

## Conclusion

In the design of biomechanical models of a myotendinous apparatus for the prediction of joint angles during limb movements, an exact separation between muscle and tendon length is necessary. If the tendon length (i.e. tendon displacement from resting length) was estimated by means of a tendon model, the muscle length could be calculated. It is important that the tendon model includes a non linear toe region for small forces. Existing work used biomechanical data of tendon stiffness from anatomical cadaver or *in vitro* studies [1, 19, 21], whose transfer ability to living systems can be discussed in general. The present work proposed an experimental setup in which the *biceps brachii* distal myotendinous junction displacement is observed for different elbow angles at varying wrist forces (muscle contraction) *in vivo*. The myotendinous junction displacement in a bone local coordinate system was extracted from ultrasonic B-mode videos using the proposed manual and (semi-) automatic method. This myotendinous junction displacement was then used together with the wrist force and a two dimensional tendon path model to fit a non linear stiffness model motivated by the experiments of [2]. The two dimensional tendon path model showed good agreement with aspects of *in vitro* measurements (see Fig 14A). Due to the assumption, that the *biceps brachii* is the only active muscle, discussed in the previous section, the existing tendon model can not be used to extract the stiffness of the tendon. It is however suitable, for the prediction of *biceps brachii* tendon displacement at a given elbow torque for a given elbow angle sub-range. The fitted tendon model conformed with linear stiffness measurements by [10], who applied the same assumptions as in this work. Compared to [10], the presented tendon model is more suitable for the prediction of myotendinous junction displacement at small elbow torques than those models that only use a linear stiffness curve defined by two points (20% and 80% of the maximum voluntary force, [10]).

## Supporting information

S1 Table Anatomical measurements distances between anatomical landmarks. (PDF)

S2 Table Bone line detection Canny-Edge-Detection parameters as in the OpenCV python package opencv-python version 4.5.2.54 at PyPI. (PDF)

S3 Table Bone line detection Hough-Line-Transformation parameters as in the OpenCV python package opencv-python version 4.5.2.54 at PyPI. (PDF)

S4 Table Bone line detection DBSCAN parameters as in the SciPy python package scipy version 1.6.3 at CONDA-FORGE. (PDF)

S5 Table Fitted model parameters model parameters for the fits in Figs 12, 13 for each subject. (PDF)

S6 Table Elbow angle dependent lever arms the distance between the elbow joint and wrist strap depends on the elbow angle (cmp. Eq (33)). Here this distance is listed for each subject. (PDF)
