# Supplementary material for: Manual and semi-automatic determination of elbow angle-independent parameters for a model of the biceps brachii distal tendon based on ultrasonic imaging
Source: PLoS One. 2022 Oct 6;17(10):e0275128. doi: 10.1371/journal.pone.0275128 (PMC9536606; doi:10.1371/journal.pone.0275128)
Supplement: S1 Table — (PDF) [file pone.0275128.s001.pdf]

S1 Table. Anatomical measurements Distances between anatomical landmarks.

quantity		subject		
symbol	description	0	1	2
$l_{a,e}$ in mm	*acromium* to *epicondyle*	330	335	310
l_{cpc} in mm	*claviala processe* to elbow crook	295	360	320
d_u in mm	largest diameter right upper arm	297	338	285
d_l in mm	largest diameter forearm	287	305	265
l_u in mm	length of the *ulna*	280	325	296
l_r in mm	length of *radius*	262	295	227
l_{cphp} in mm	*claviala processe* to *humerus* projection on parasigatial plane	35	50	39
