# Supplementary material for: Manual and semi-automatic determination of elbow angle-independent parameters for a model of the biceps brachii distal tendon based on ultrasonic imaging
Source: PLoS One. 2022 Oct 6;17(10):e0275128. doi: 10.1371/journal.pone.0275128 (PMC9536606; doi:10.1371/journal.pone.0275128)
Supplement: S2 Table — (PDF) [file pone.0275128.s002.pdf]

S2 Table. Bone line detection Canny-Edge-Detection parameters as in the OpenCV python package opencv-python version 4.5.2.54 at PyPI.

symbol in text	function parameter	value
$\mathrm{grad}_{\mathrm{min}}$	threshold1	50
$\mathrm{grad}_{\mathrm{max}}$	threshold2	220
a_s	apertureSize	3
	L2gradient	False
