# Supplementary material for: Manual and semi-automatic determination of elbow angle-independent parameters for a model of the biceps brachii distal tendon based on ultrasonic imaging
Source: PLoS One. 2022 Oct 6;17(10):e0275128. doi: 10.1371/journal.pone.0275128 (PMC9536606; doi:10.1371/journal.pone.0275128)
Supplement: S3 Table — (PDF) [file pone.0275128.s003.pdf]

S3 Table. Bone line detection Hough-Line-Transformation parameters as in the OpenCV python package opencv-python version 4.5.2.54 at PyPI.

symbol in text	function parameter	value
Δr	rho	1
$\Delta \varphi$	theta	$\frac{\pi}{180}$
$\mathrm{Threshold}_{\mathrm{Hough}}$	threshold	40
