# Supplementary material for: Manual and semi-automatic determination of elbow angle-independent parameters for a model of the biceps brachii distal tendon based on ultrasonic imaging
Source: PLoS One. 2022 Oct 6;17(10):e0275128. doi: 10.1371/journal.pone.0275128 (PMC9536606; doi:10.1371/journal.pone.0275128)
Supplement: S4 Table — (PDF) [file pone.0275128.s004.pdf]

S4 Table. Bone line detection DBSCAN parameters as in the SciPy python package scipy version 1.6.3 at CONDA-FORGE.

symbol in text	function parameter	value
ϵ_{core}	eps	0.02
$n_{\mathrm{min,s}}$	min_samples	2
