# Supplementary material for: Manual and semi-automatic determination of elbow angle-independent parameters for a model of the biceps brachii distal tendon based on ultrasonic imaging
Source: PLoS One. 2022 Oct 6;17(10):e0275128. doi: 10.1371/journal.pone.0275128 (PMC9536606; doi:10.1371/journal.pone.0275128)
Supplement: S5 Table — (PDF) [file pone.0275128.s005.pdf]

S5 Table. Fitted model parameters Model parameters for the fits in Figs. 12 and 13 for each subject.

Θ_l	experiment method	$\vec{\rho}$ c_1	c_2	Δl_{toe}
	**subject 0**			
30	manual	832.07	3922.14	0.01202
30	(semi-) automatic	804.28	1944.93	0.01220
45	manual	977.99	1330.04	0.00494
45	(semi-) automatic	573.69	2629.44	0.01325
60	manual	571.16	3164.89	0.01273
60	(semi-) automatic	562.84	2069.93	0.01264
75	manual	611.97	1623.51	0.01433
75	(semi-) automatic	657.34	837.64	0.01508
	**subject 1**			
30	manual	215.83	25203.50	0.02016
30	(semi-) automatic	261.59	24976.83	0.01315
45	manual	179.08	24423.94	0.01479
45	(semi-) automatic	206.27	24606.29	0.01244
60	manual	133.40	26381.40	0.01760
60	(semi-) automatic	138.26	29294.39	0.01325
75	manual	130.58	23854.13	0.02164
75	(semi-) automatic	273.28	17475.15	0.00649
	**subject 2**			
30	manual	607.17	609.76	0.01580
30	(semi-) automatic	367.42	3265.08	0.01522
45	manual	502.98	890.98	0.01549
45	(semi-) automatic	322.10	3379.15	0.01601
60	manual	438.33	1056.30	0.01423
60	(semi-) automatic	247.67	4449.44	0.02066
75	manual	3099.03	0.00	0.00711
75	(semi-) automatic	195.02	4797.09	0.01806
