# Supplementary material for: Manual and semi-automatic determination of elbow angle-independent parameters for a model of the biceps brachii distal tendon based on ultrasonic imaging
Source: PLoS One. 2022 Oct 6;17(10):e0275128. doi: 10.1371/journal.pone.0275128 (PMC9536606; doi:10.1371/journal.pone.0275128)
Supplement: S6 Table — (PDF) [file pone.0275128.s006.pdf]

S6 Table. Elbow angle dependent lever arms The distance between the elbow joint and wrist strap depends on the elbow angle (cmp. eq. 33). Here this distance is listed for each subject.

subject 0

Θ in °	90	75	60	45	30	15	0
$l_w(\Theta)$ in m	0.220	0.236	0.247	0.251	0.254	0.259	0.259

subject 1

Θ in °	90	75	60	45	30	15	0
$l_w(\Theta)$ in m	0.214	0.229	0.239	0.244	0.249	0.254	0.257

subject 2

Θ in °	90	75	60	45	30	15	0
$l_w(\Theta)$ in m	0.237	0.244	0.253	0.256	0.261	0.264	0.277
